# Supplementary material for: Time-resolved oxidative signal convergence across the algae–embryophyte divide
Source: Nat Commun. 2025 Feb 19;16:1780. doi: 10.1038/s41467-025-56939-y (PMC11840003; doi:10.1038/s41467-025-56939-y)
Supplement: Supplementary file 1 — Supplementary Information [file 41467_2025_56939_MOESM1_ESM.pdf]

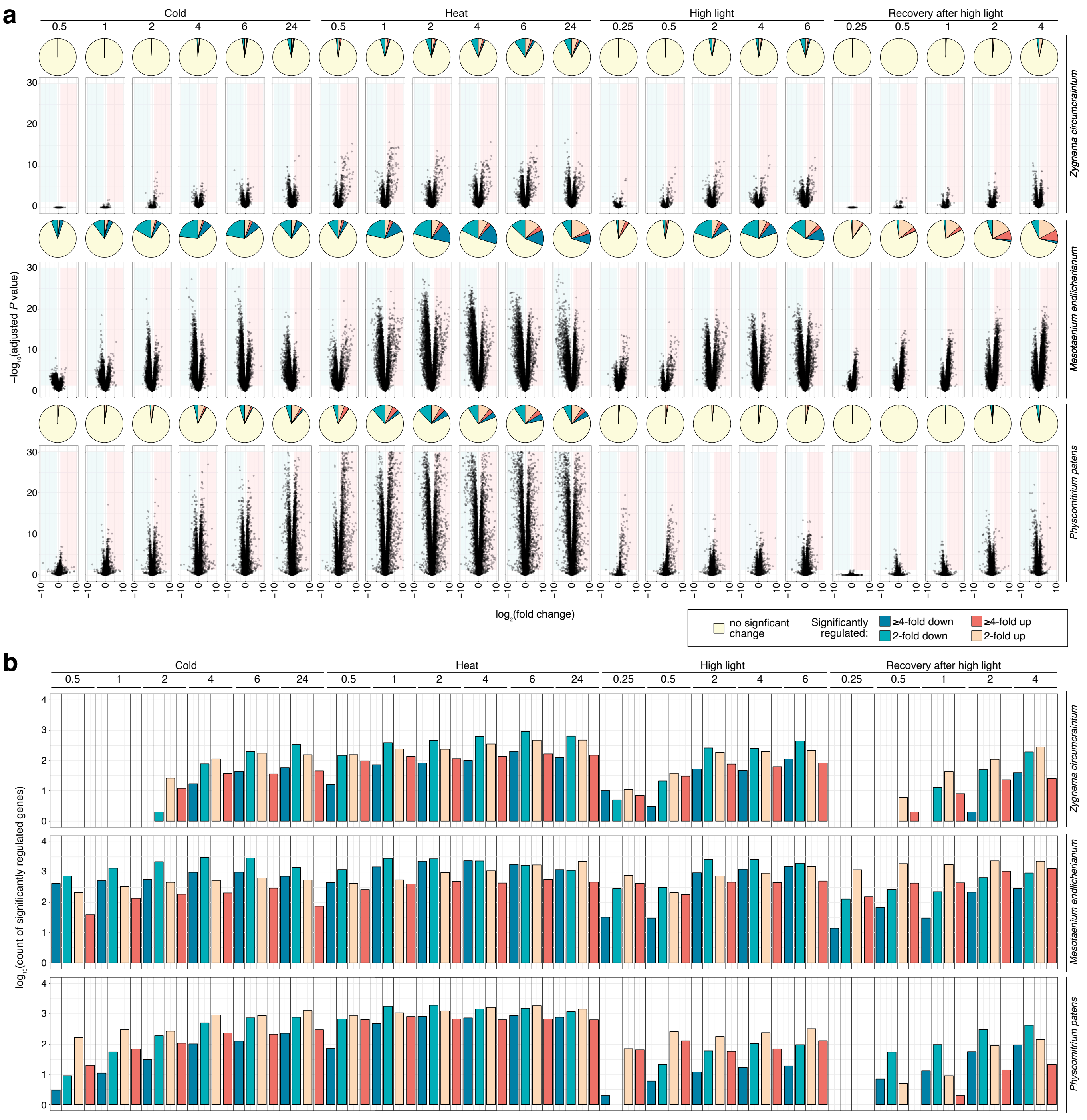

Supplementary Figure 1: Differential gene expression comparisons highlight heat responsiveness. Significance of gene expression change as $\log_2$(fold change) due to stress exposure in all three species. (a) Pie diagrams of the respective portion of 2-fold up to ≥4-fold up and downregulated genes and corresponding Volcano plots with Benjamini-Hochberg adjusted *P*-value ($\log_{10}$-scaled). (b) Bar plots of $\log_{10}$ adjusted counts of the number of significantly 2-fold and ≥4-fold up and downregulated genes. Statistics build on modelling gene expression changes under each treatment samples compared to the same time point in the control condition using limma.

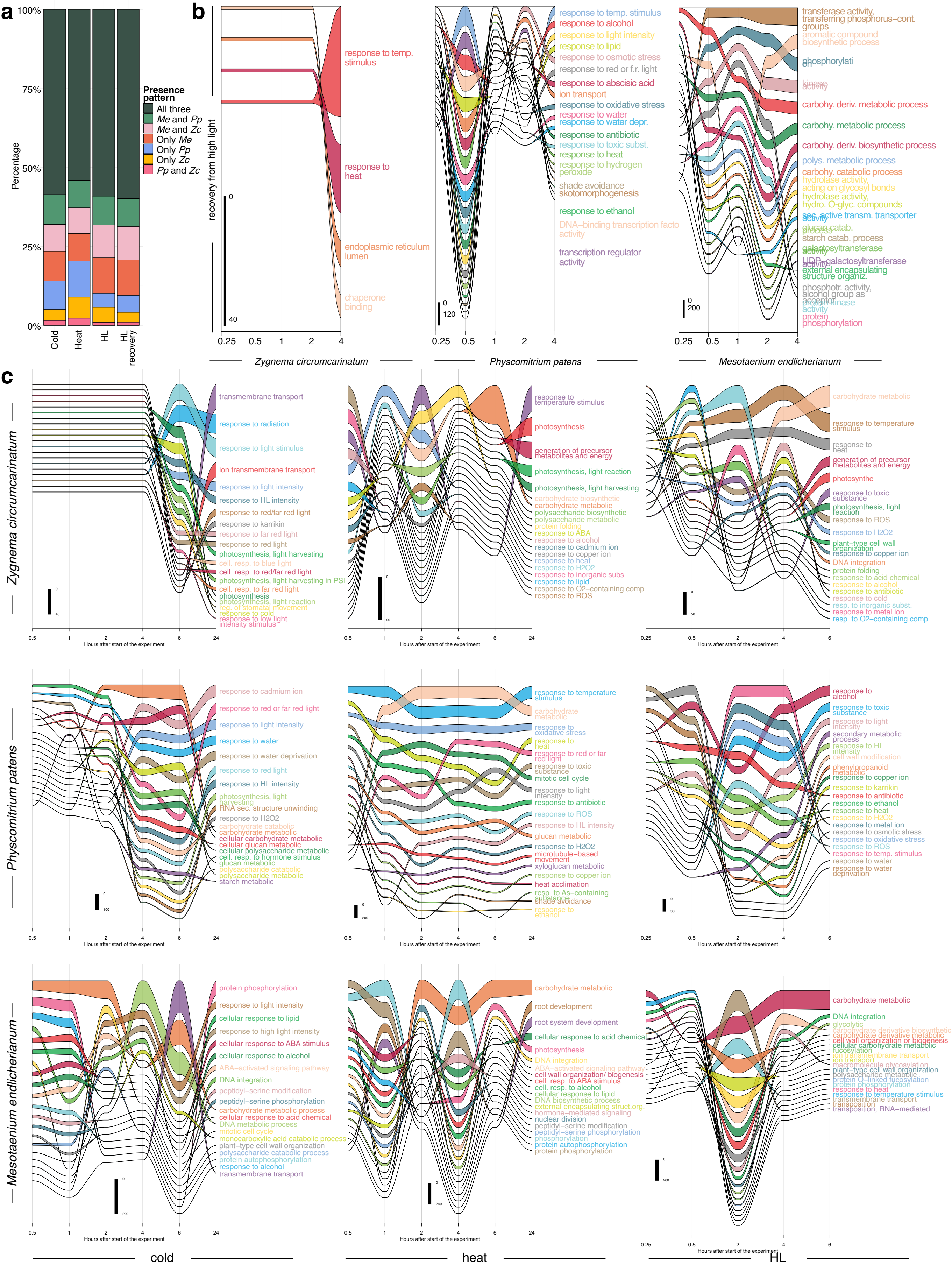

Supplementary Figure 2: (a) Distribution of differentially expressed genes in the four treatments across species based on assignments to HOGs. **(b)** Alluvial plots on GO terms associated with high light recovery. Time-resolved (in hours after start of the experiment) alluvial diagrams of most prominent GO terms principally conserved in all three species with all associated differentially regulated genes (number indicated by line width) during high light recovery under control conditions, category time scale. Note the delayed responses in *Zygnema circumcarinatum*. **(c)** Time-resolved alluvial diagrams of most prominent GO terms from the category "Biological Process" found across at least three time points based on significantly differentially regulated genes (term size indicated by line width) during cold, heat and high light stress exposure, category time scale.

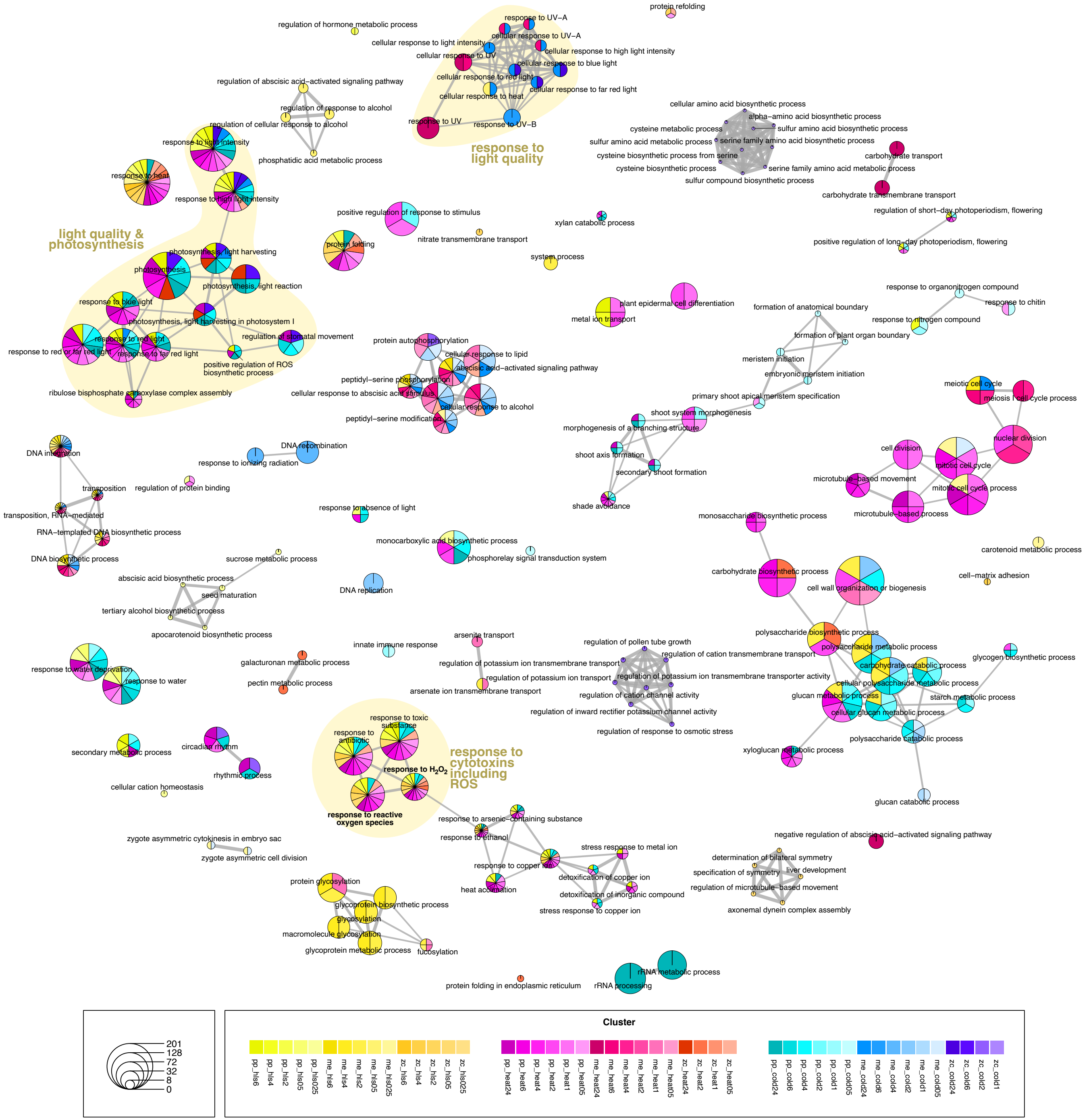

Supplementary Figure 3: Biological theme comparison of GO terms pinpointed by significantly regulated genes. Most enriched GO terms among the significantly differentially expressed genes in 51 comparisons (stress versus control) represented by color code in all three organisms and their relative enrichment shown by pie slice size and number of genes per GO term represented by circle size of pie plots.

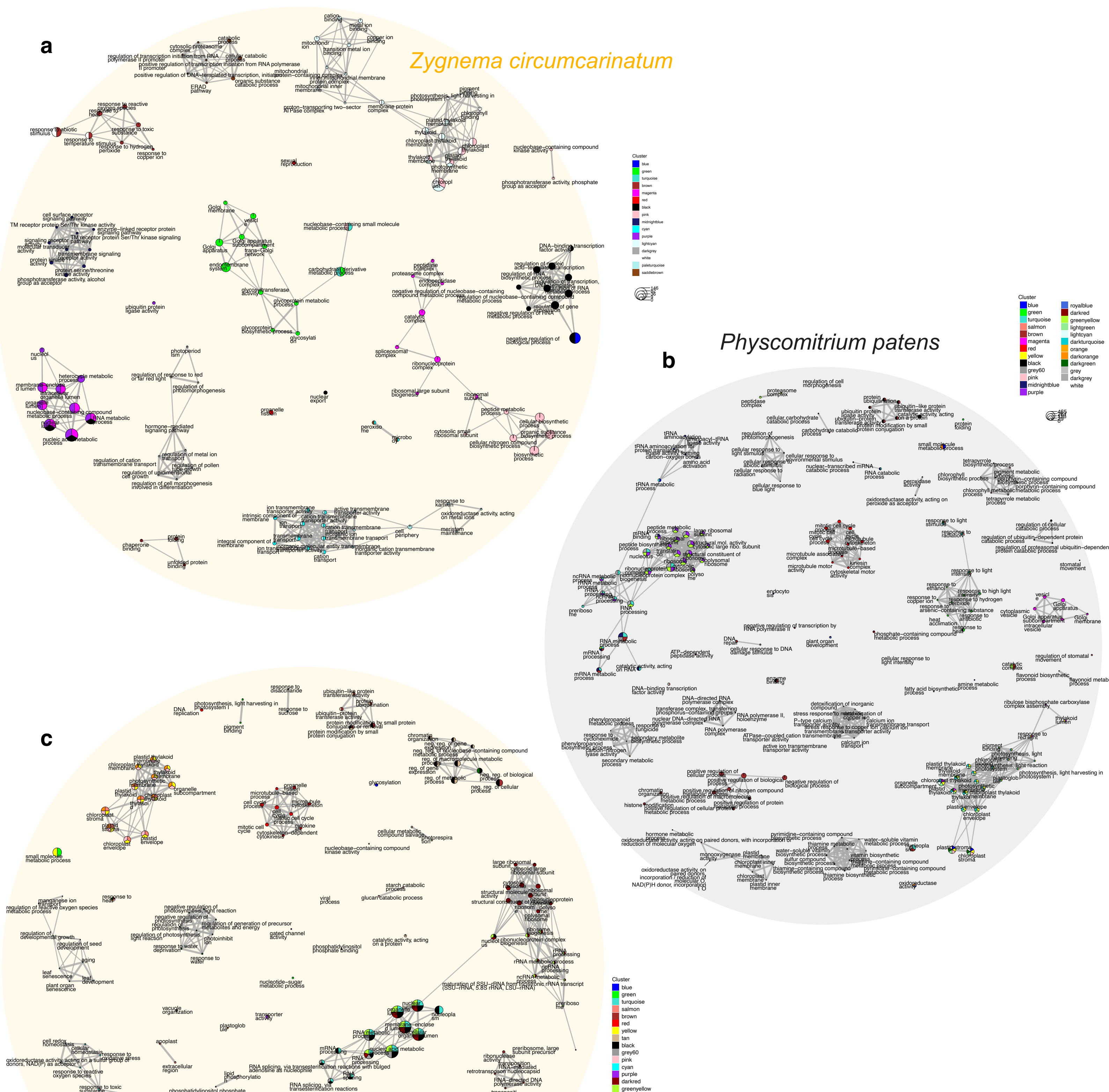

Supplementary Figure 4: Biological theme comparison of GO terms associated with modules recovered by WGCNA. Color coded comparison of clusters of most enriched GO terms in respective WGCNA clusters in a *Zygnema circumcarinatum, Physcomitrium patens,* and *Mesotaenium endlicherianum*. Number of genes per enriched GO term is represented by the size of the circles.

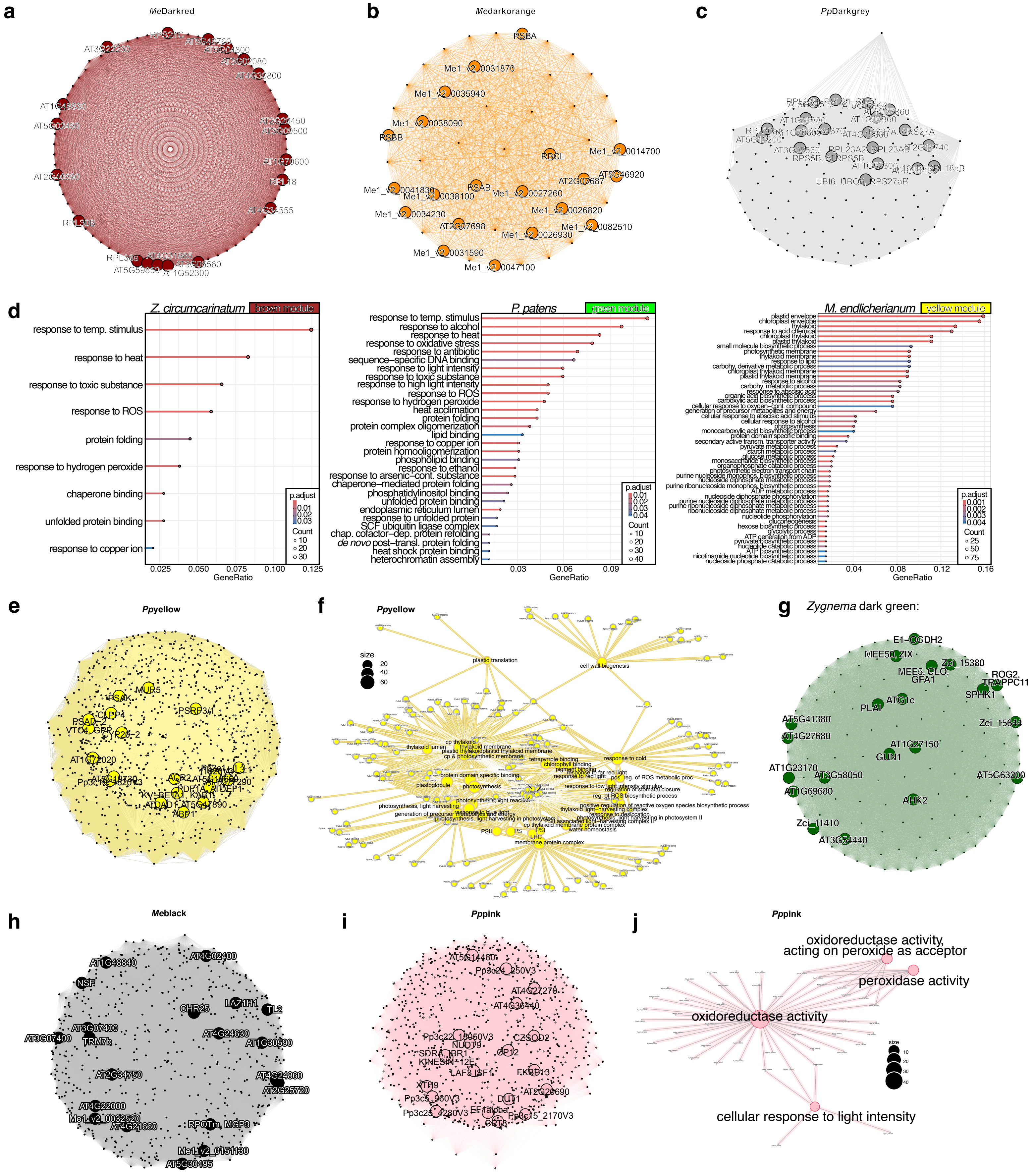

Supplementary Figure 5: GO terms and hubs in modules recovered by WGCNA. (a,b,c,e,g,h,i) Most enriched genes and GO terms in some representative WGCNA clusters from Figure 2. (d) dotplot of enriched GO terms. (f,j) Cnet plot of enriched GO terms.

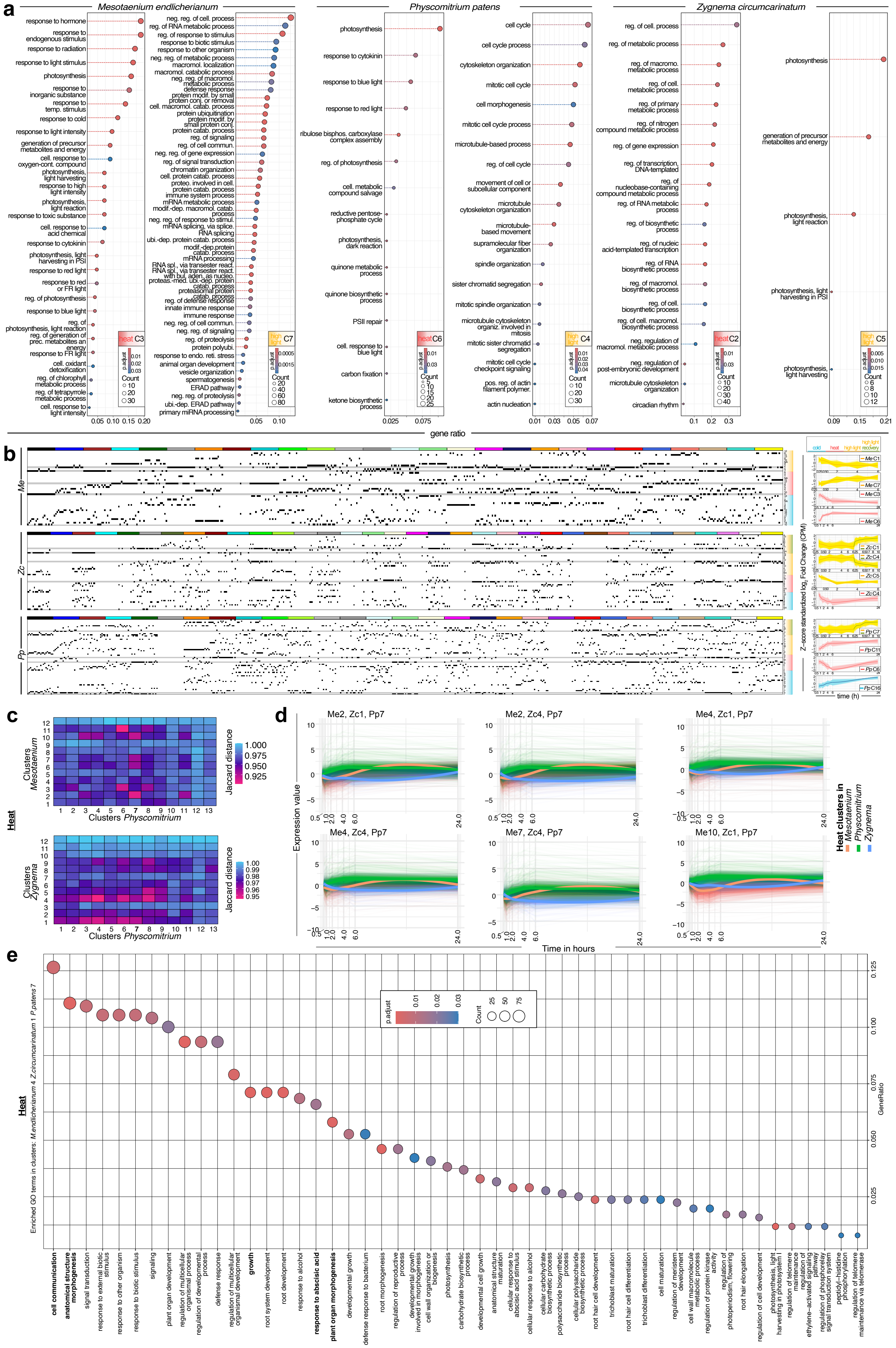

Supplementary Figure 6: Commonalities and differences gleaned from DPGP-defined temporal gene expression clusters. (a) Significantly enriched GO terms of some DPGP clusters of Figure 5 including all three species. (b) Left: Black ticks indicate into which DPGP clusters the hub genes recovered by WGCNA (indicated by colors) fall; right: line plots of the expression behavior of cohorts of genes in DPGP clusters that are particularly rich in WGCNA-defined hub genes. Grey lines mark those DPGP clusters whose expression profiles are shown on the right. (c) Most Jaccard similar clusters recovered for heat response. (d) Expression profiles of *Physcomitirum* cluster 7 upon heat (*Pp*H7) compared to its most Jaccard similar clusters in algae. (e) Example of enriched GO terms for the genes in *Me*H4, *Zc*H1, and *Pp*H7.

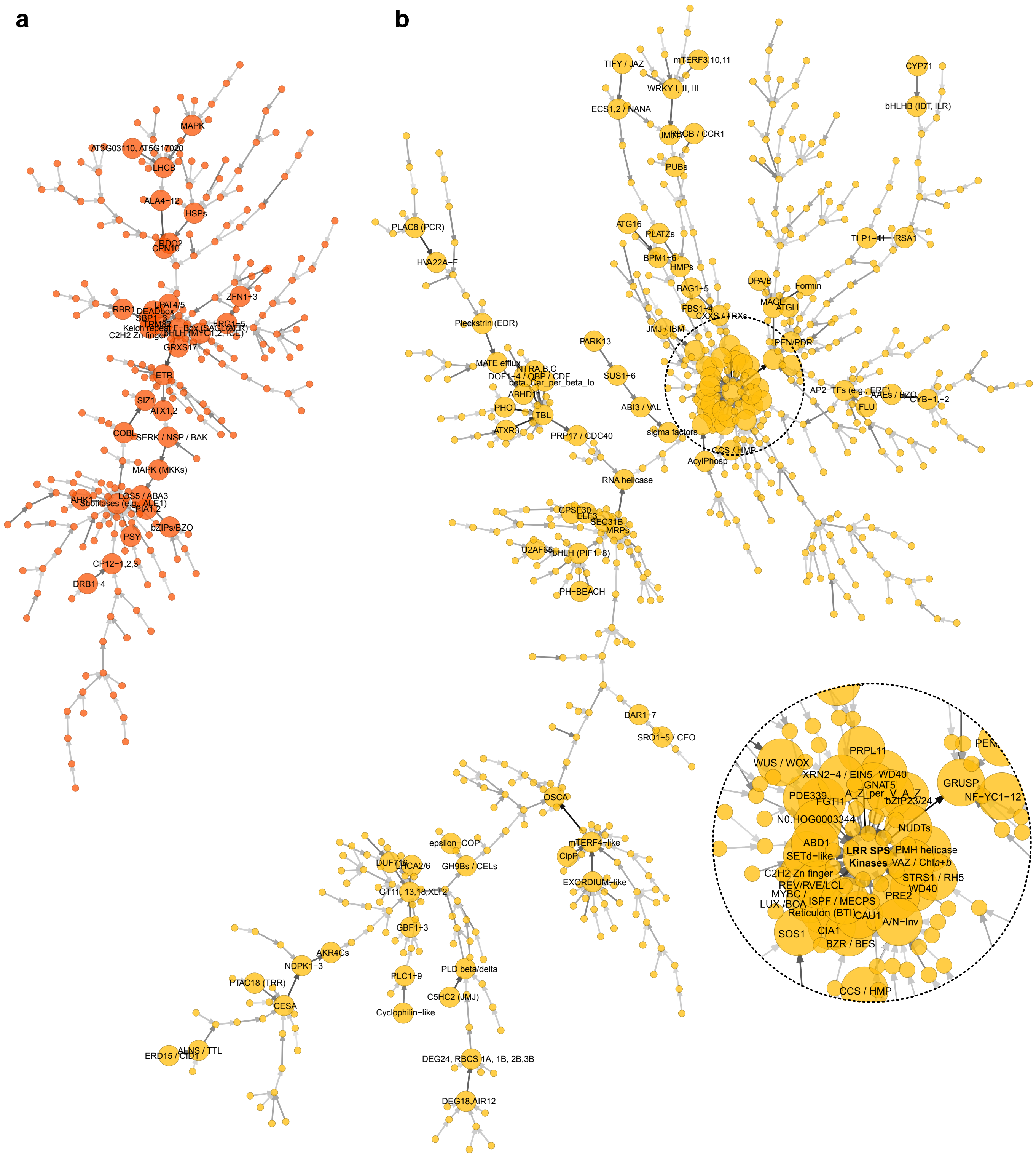

Supplementary Figure 7: All shared connections predicted across all networks inferred via SWING-RF. SWING-RF network filtered based on HOGs and metabolites conserved in all three species. Hierarchical orthogroups (HOGs) are annotated if they engage in the top 100 most predictive relationships. Two networks (a) and (b) were recovered. Inset: Zoom into the central 'kinome' (*LRR*)–*Ser/Thr kinases* hub.

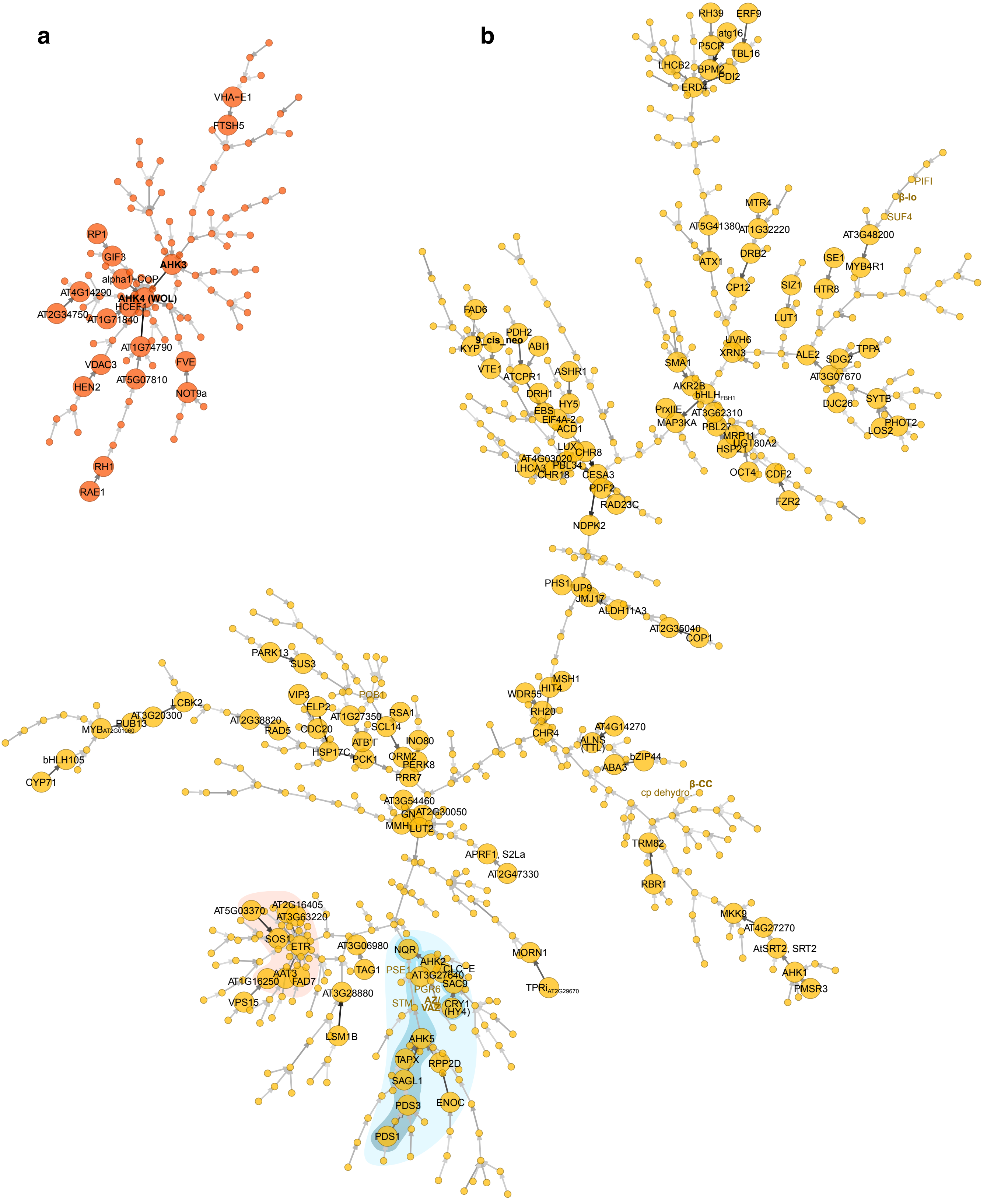

Supplementary Figure 8: Predicted gene regulatory network, via SWING-RF, annotated based on best BLAST hits (BBH). SWING-RF of 100 most predictive (enlarged circles) genes (best BLAST hit-defined homologs) and metabolites conserved in all three species. Two networks (a and b) were recovered: (a) an ‘AHK centered’ cluster; (b) Central stress responsive cluster resembling some important parts of Figure 6. Homologs are annotated if they engage in the top 100 most predictive relationships.

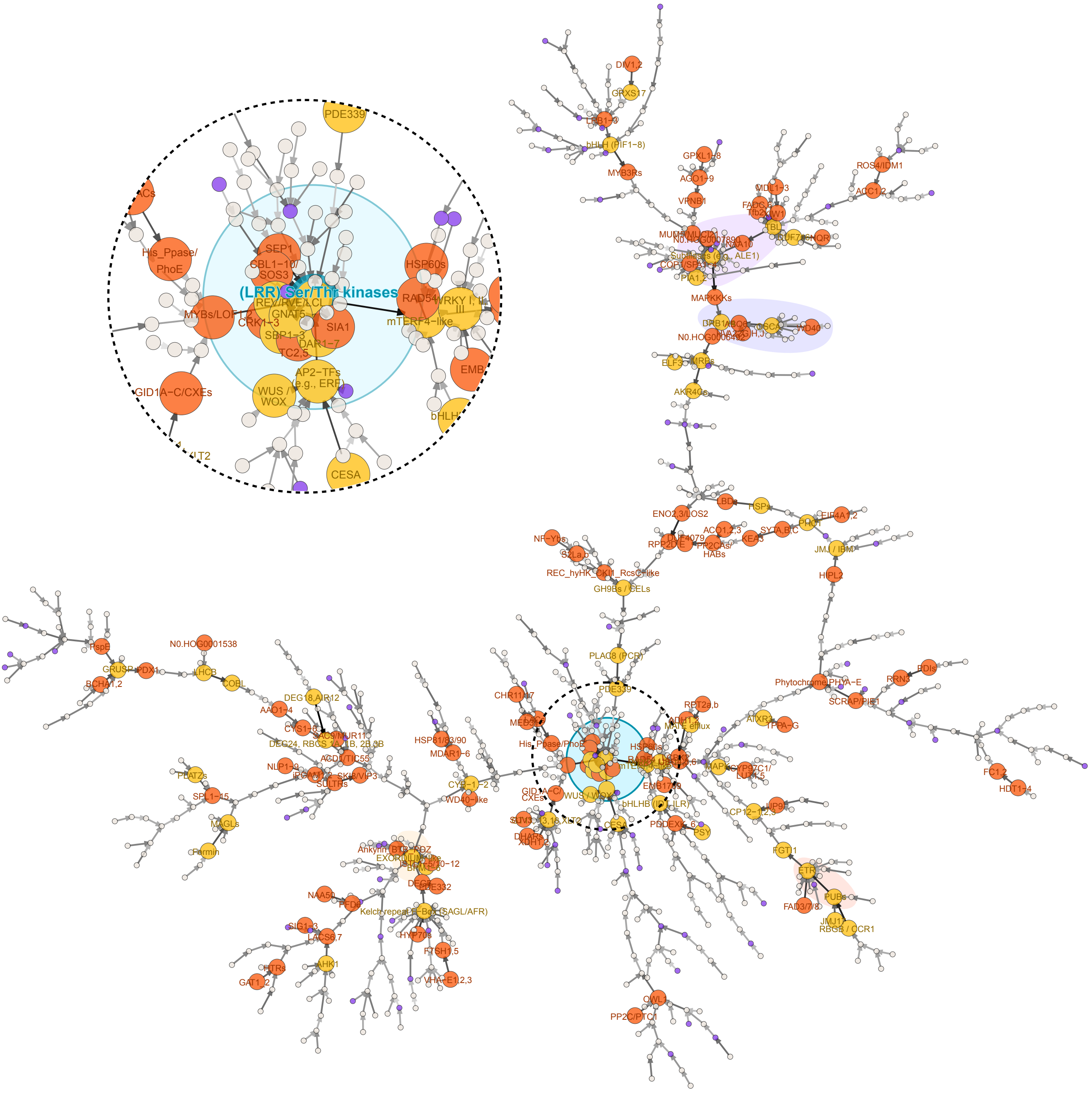

Supplementary Figure 9: Predicted gene regulatory network, via SWING-RF, computed with all time points for which transcriptome data were generated. A predicted gene regulatory network of 900 nodes and edges. HOGs are annotated if they engage in the top 100 most predictive relationships across all three species. Yellow nodes are genes that have been recovered in both the SWING networks (i.e. with all time points and only those time points for which also metabolite data were generated, see Figure 6 and Supplementary Figures 7 and 8). Orange nodes are genes that, upon recomputing the network with all time series data, became some of the 100 most predictive. Purple indicates genes that were among the top 100 most predictive genes in the networks that included the metabolite data but are now among the ranks 800 to 900. Grey nodes are genes that were in both predictions not in the top 100. Inset: Zoom into the central 'kinome' (*LRR*)–*Ser/Thr kinases* hub.

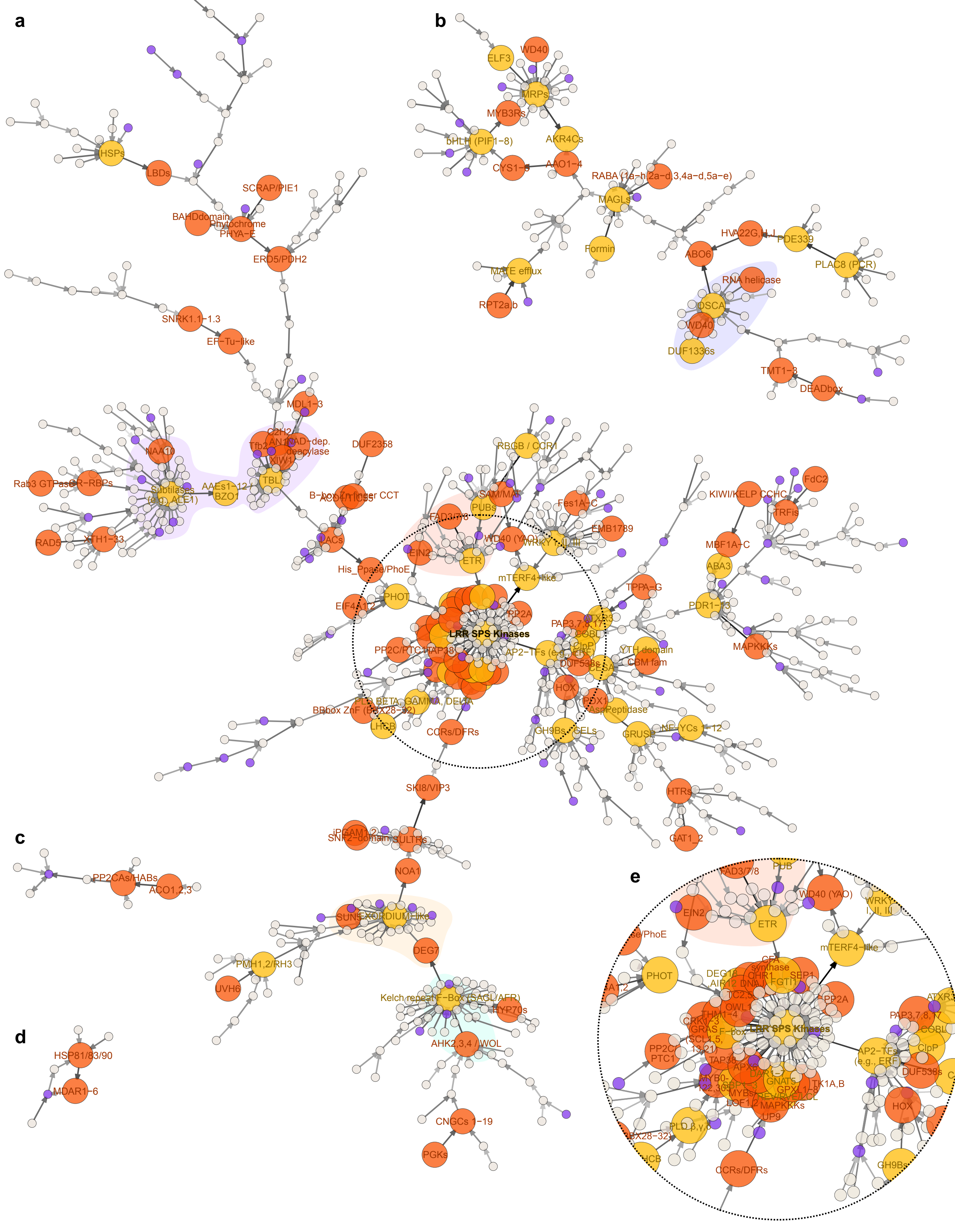

Supplementary Figure 10: Predicted gene regulatory network, re-computed with all time points for which transcriptome data were generated (without metabolite data) and filtered based on conserved DPGP clusters. A predicted gene regulatory networks (a) to (d) of 900 nodes and edges. HOGs are annotated if they engage in the top 100 most predictive relationships across all three species. Yellow nodes are genes that have been recovered in both the SWING networks (i.e. with all time points and only those time points for which also metabolite data were generated, see Figure 6 and Supplementary Figures 7 and 8). Orange nodes are genes that, upon recomputing the network with all time series data, became some of the 100 most predictive. Purple indicates genes that were among the top 100 most predictive genes in the networks that included the metabolite data but are now among the ranks 800 to 900. Grey nodes are genes that were in both predictions not in the top 100. Four networks, (a) to (d), were recovered. (e) Zoom into the central ‘kinome’ (*LRR*)–*Ser/Thr kinases* hub.

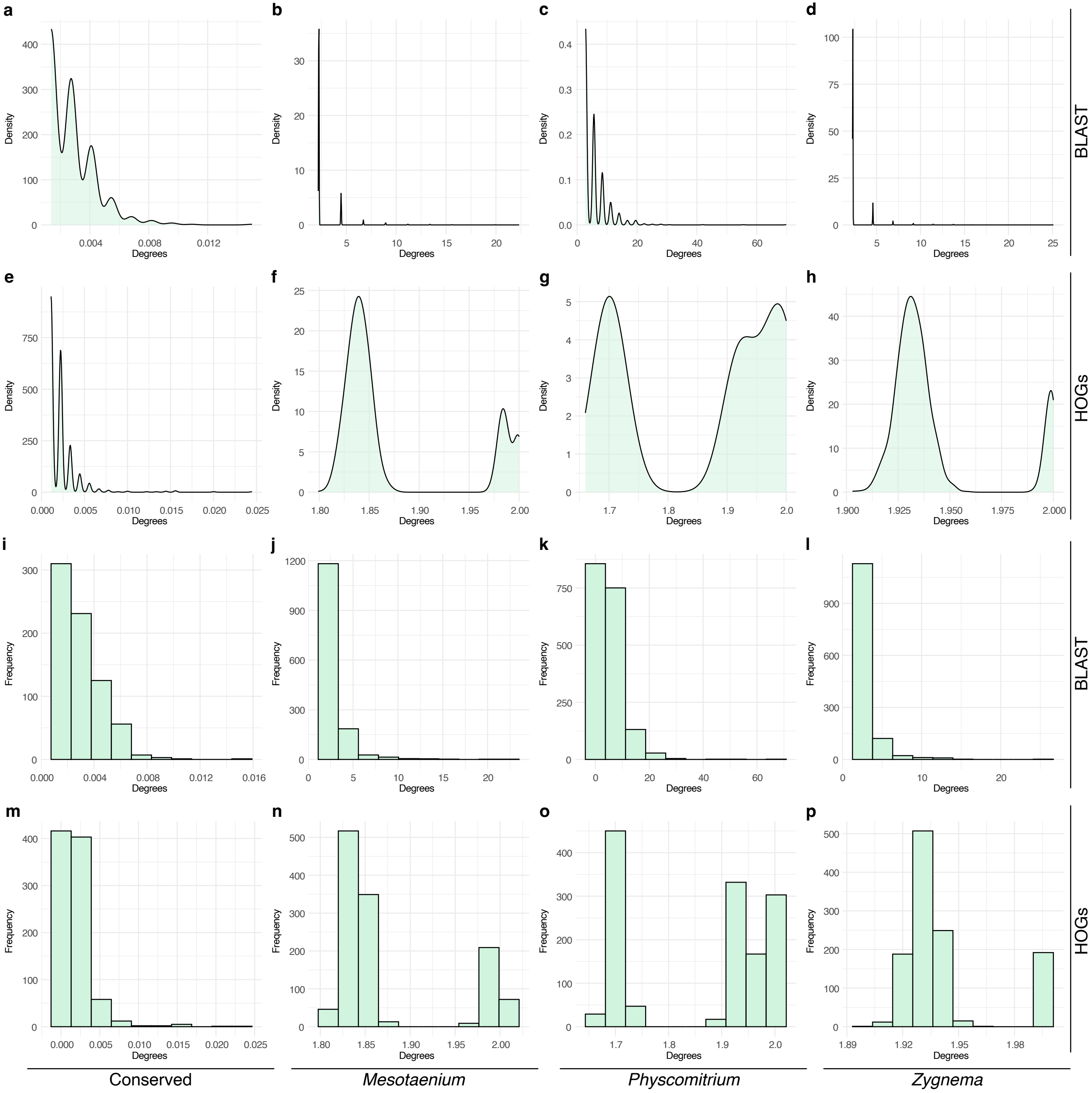

Supplementary Figure 11: Degree distribution in the networks computed by SWING-RF. Plots show the degree distribution of the networks across all three species and the combined conserved network, both the networks based on BLAST and HOGs; density plots (eight plots at the top) and histograms (eight plots at the bottom).

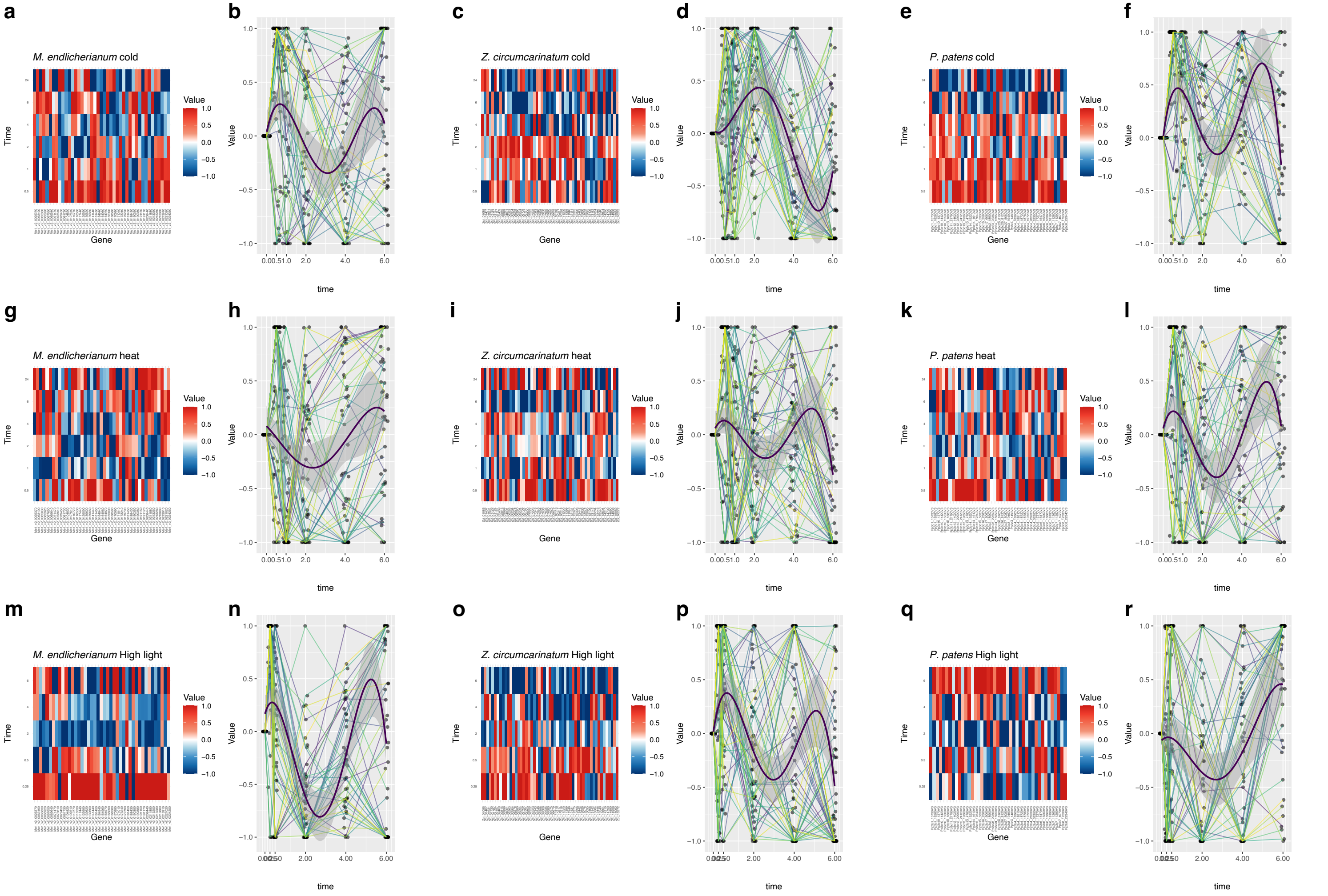

Supplementary Figure 12: Expression dynamics of all genes in the hub of *Ser/Thr kinase* genes. Expression changes of all genes clustered in the HOG that constitutes the major *Ser/Thr kinase* hub for all three species, the conditions cold (a-f), heat (g-l), and high light (m-r) relative to control.

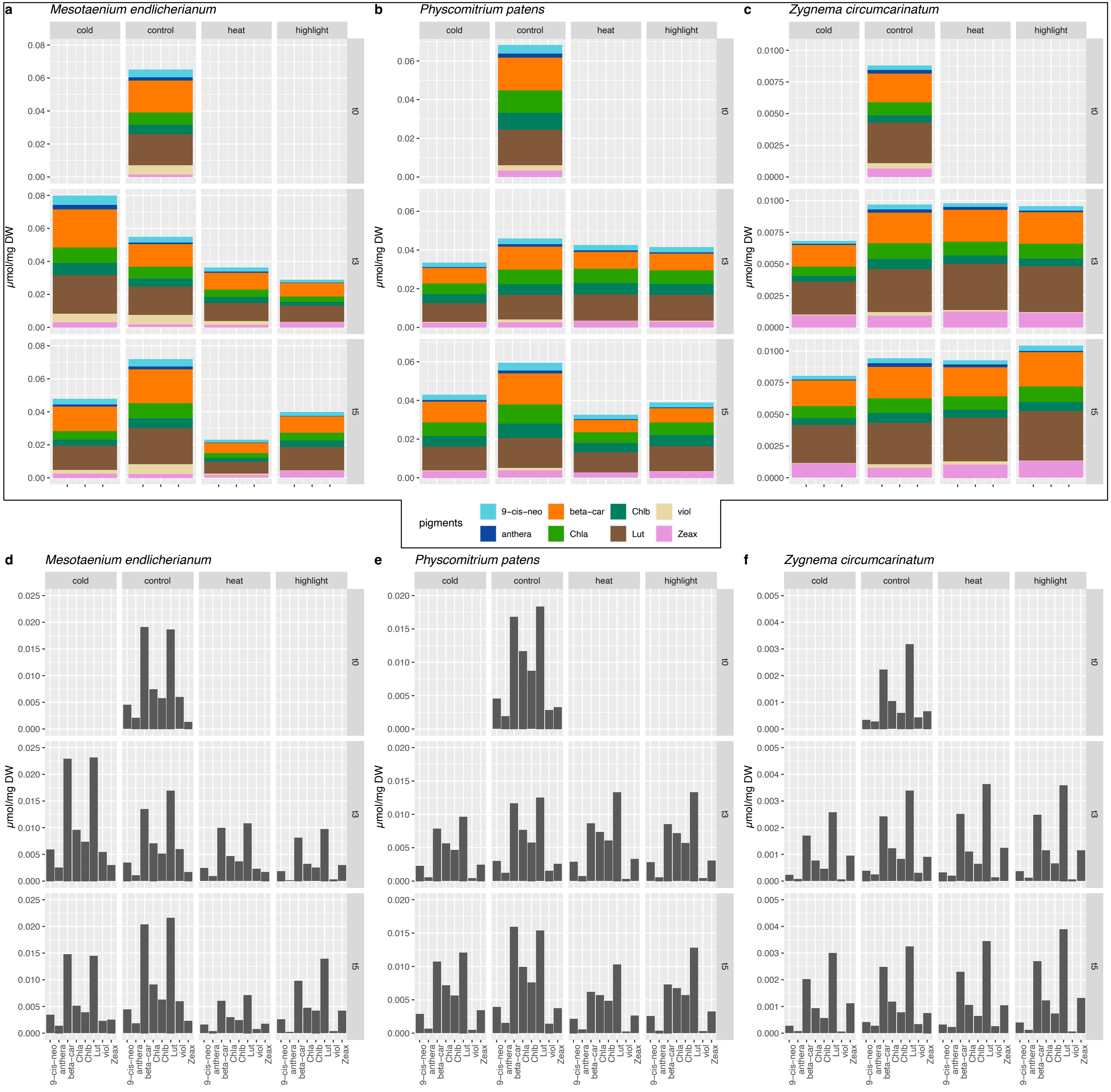

Supplementary Figure 13: Pigment pool fluctuations due to stress exposure. Selected time points of stress exposure including control, cold, heat, and high light time-series: t0 = 0 h; t3 = 2 h; t5 = 6 h. All metabolite levels in mol/mg DW. Left: *Mesotaenium endlicherianum* (a,d); Middle: *Physcomitrium patens* Gransden 2004 (b,e); Right: *Zygnema circumcarinatum* (c,f). (a-c) Pigment pool sizes (cumulative metabolite levels). (d-f) Individual metabolite levels. 9-*cis*-neo, 9-*cis*-Neoxanthin; beta-car, -Carotene; Chlb, Chlorophyll *b*; viol, Violaxanthin; anthera, Antheraxanthin; Chla, Chlorophyll *a*; Lut, Lutein; Zeax, Zeaxanthin.

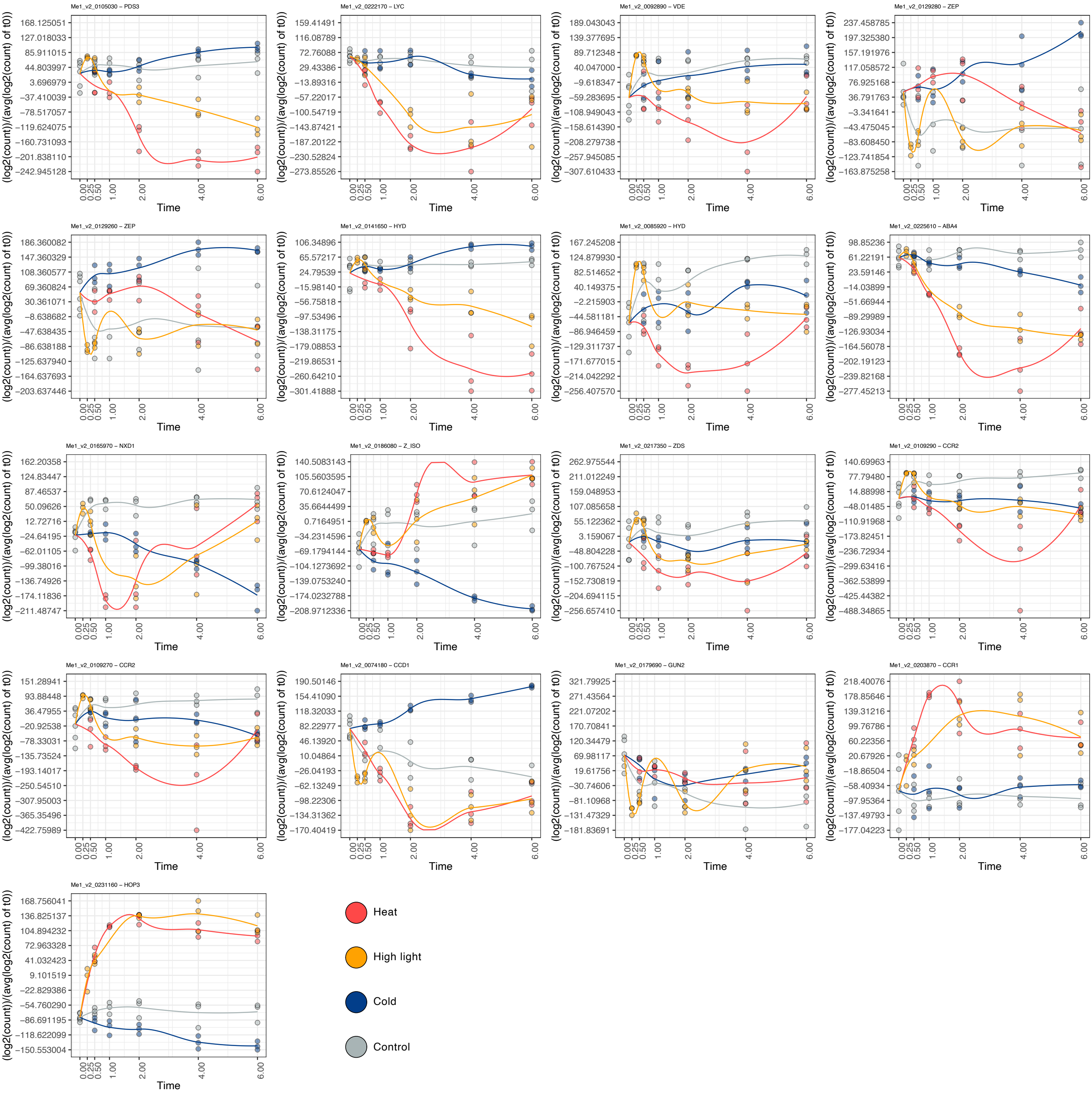

Supplementary Figure 14: Dynamics in gene expression of *Mesotaenium endlicherianum* SAG 12.97 homologs likely acting in carotenoid and apocarotenoid metabolism upon stress. Expression changes of important homologous genes likely salient to carotenoid metabolism in the first 6 h of stress exposure.

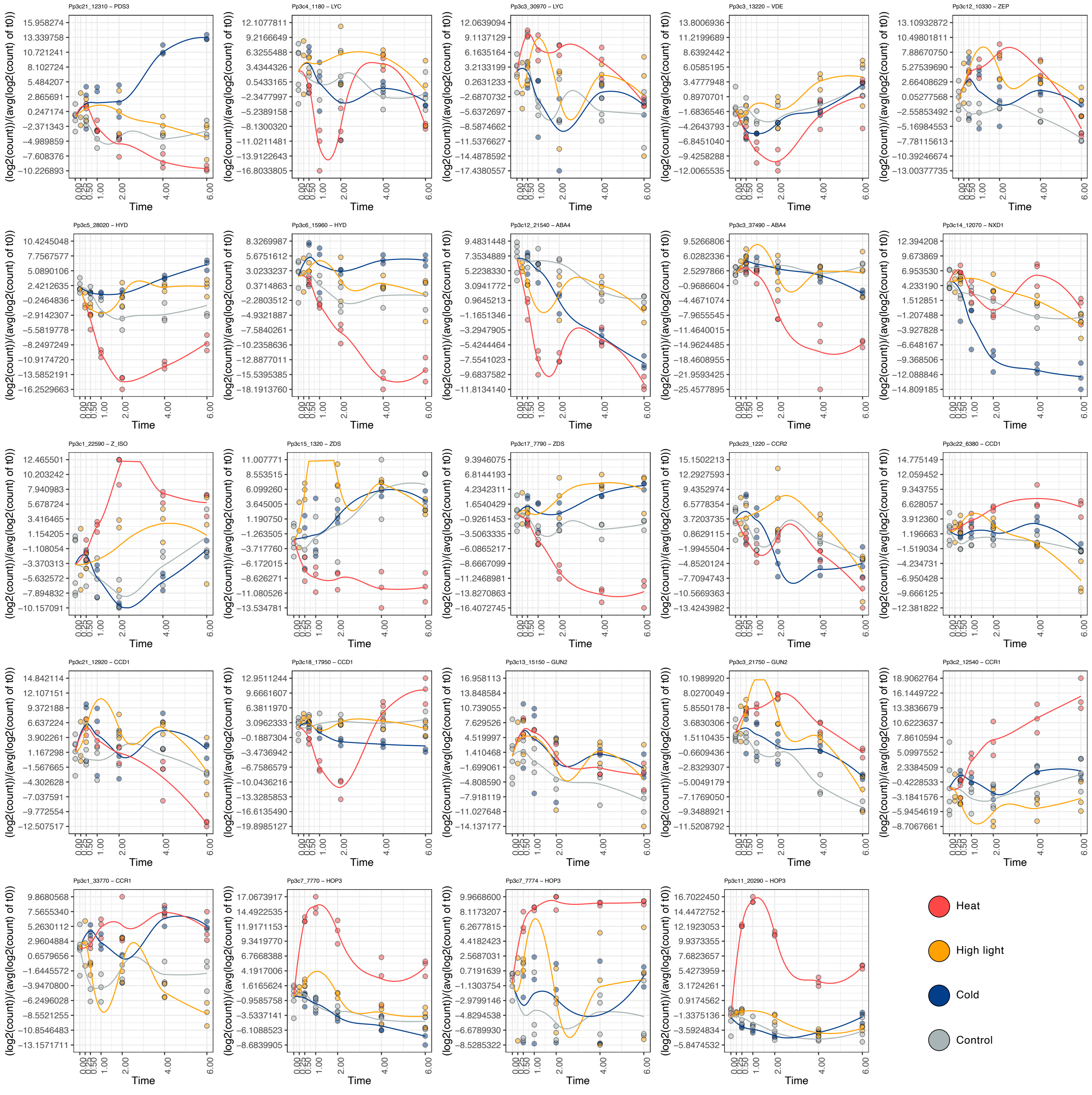

Supplementary Figure 15: Dynamics in gene expression of *Physcomitrium patens* homologs likely acting in carotenoid and apocarotenoid metabolism upon stress. Expression changes of important homologous genes likely salient to carotenoid metabolism in the first 6 h of stress exposure.

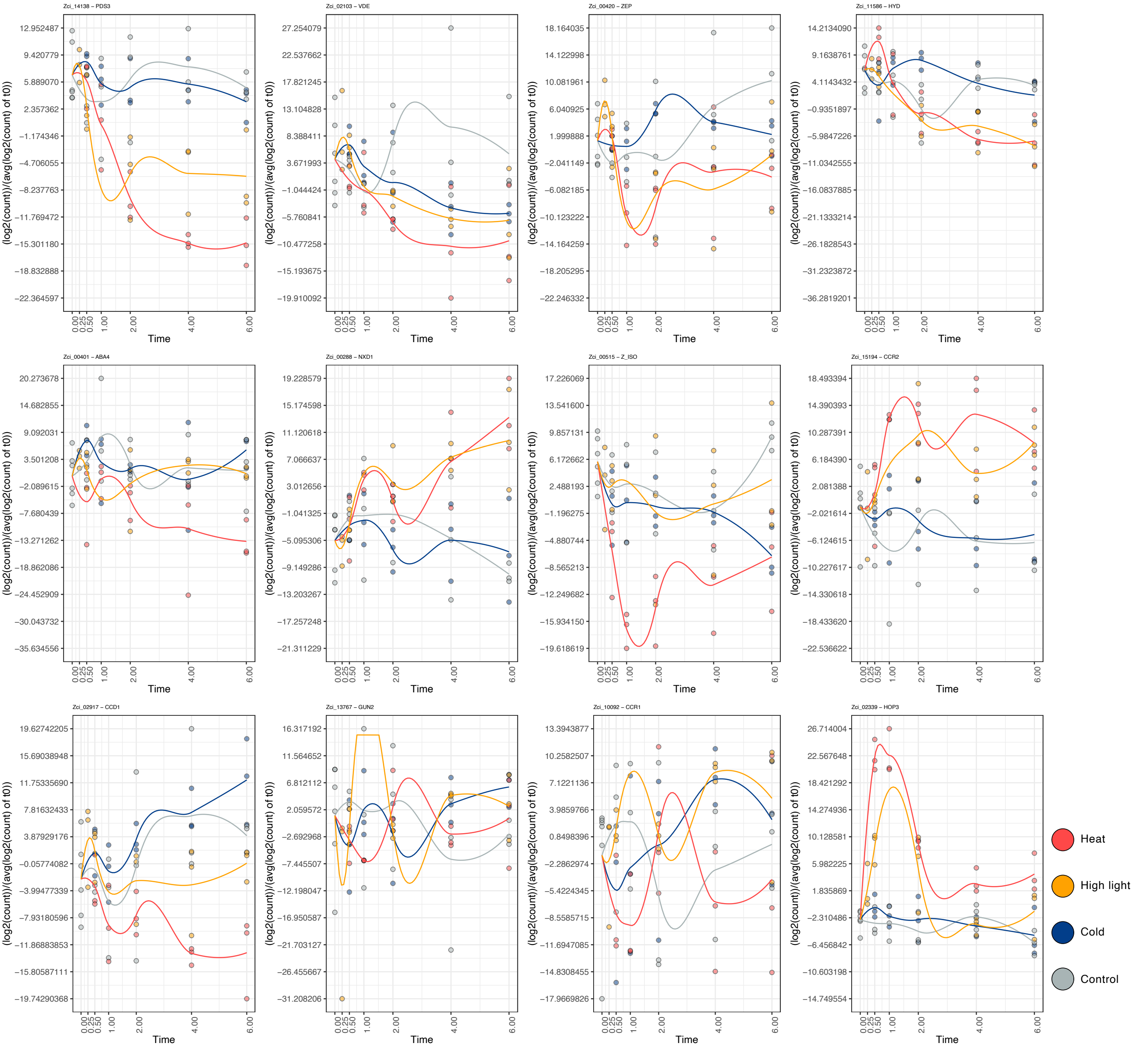

Supplementary Figure 16: Dynamics in gene expression of *Zygnema circumcarinatum* SAG 698-1b homologs likely acting in carotenoid and apocarotenoid metabolism upon stress. Expression changes of important homologous genes likely salient to carotenoid metabolism in the first 6 h of stress exposure.

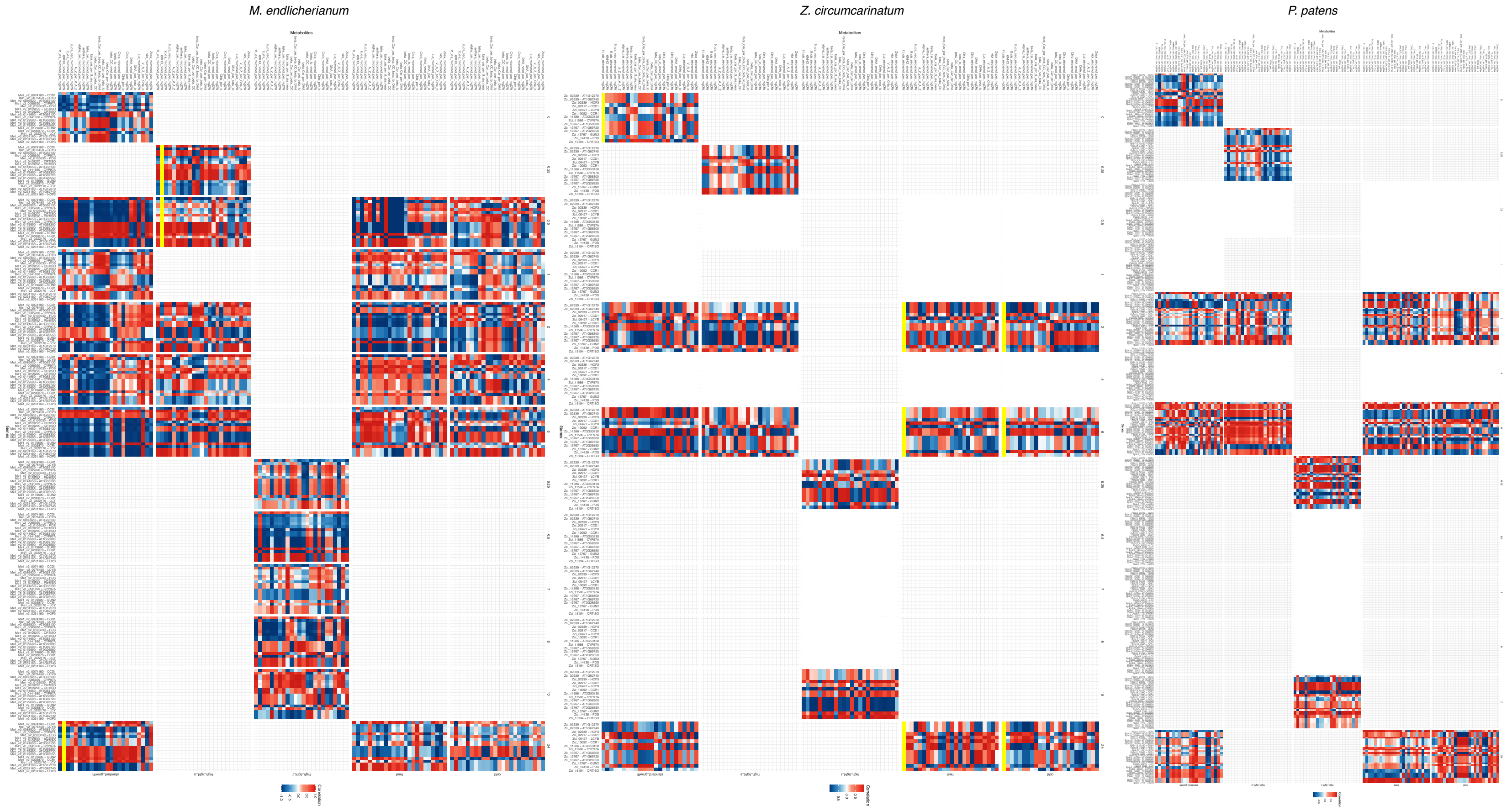

Supplemenatary Figure 17: Correlation between genes and pigments. There are four layers of information as follows: y-axis left = genes; x-axis top = amount and relative relations of pigments; grid y-axis right = time; grid x-axis bottom = treatment. Yellow means n/a. Correlation is shown from strong postive (red) to strong negative (blue).

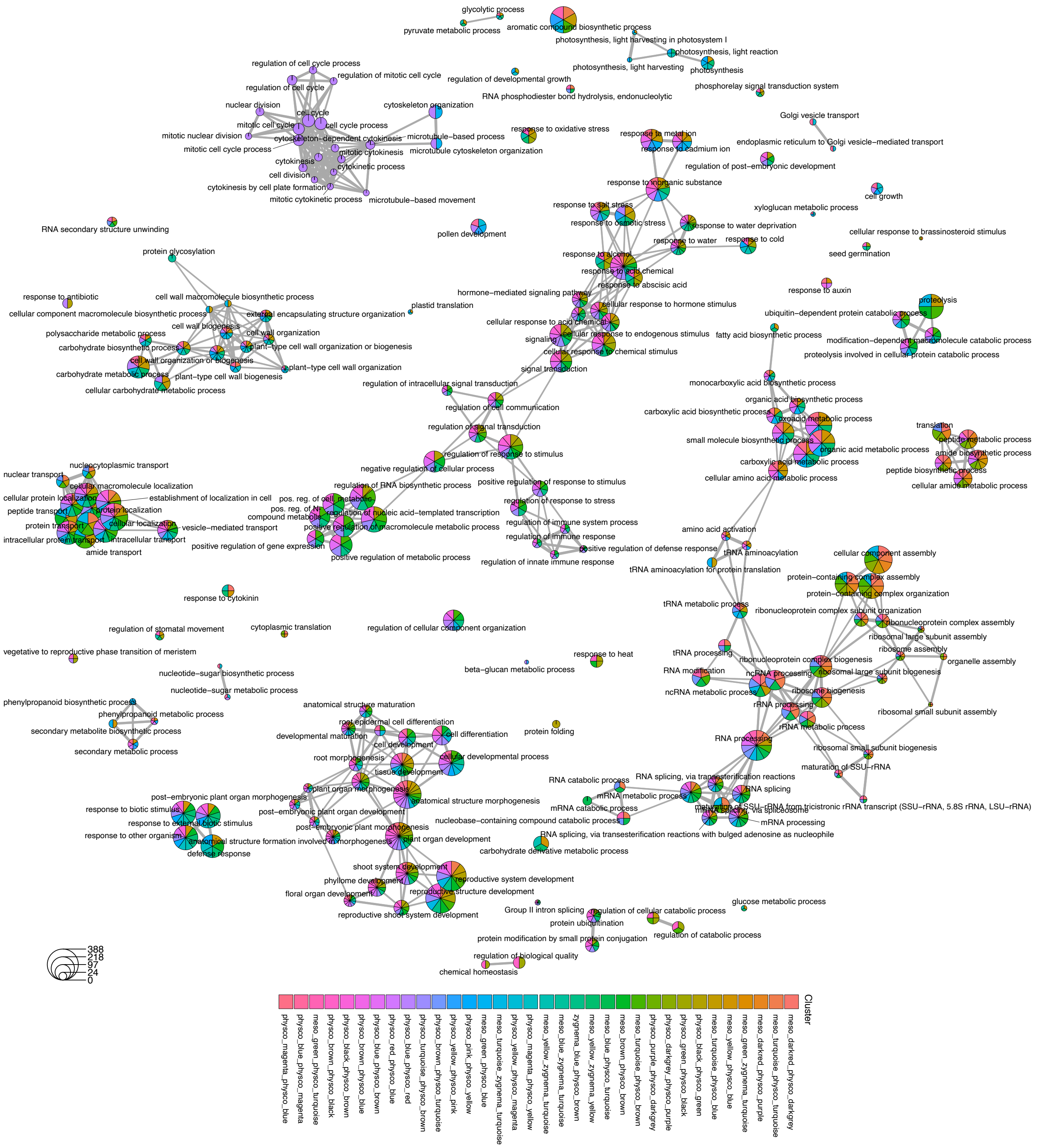

Supplementary Figure 18: Biological theme comparison of GO terms between similar WGCNA clusters. Most enriched GO terms among the clusters determined by WGCNA and found highly Jaccard similar. Different colors show different pairwise comparisons of clusters and the relative enrichment of GO terms in those two clusters is shown by pie slice size and number of genes per GO term represented by circle size of pie plots.

SUPPLEMENTARY METHODS

HPLC-UV-Vis-DAD

Method optimization

Extraction of and separation of pigments

The extraction protocol was originally inspired by Aronsson et al 2008. First (as in Aronsson et al 2008) extraction was tested with fresh weight but resulted in chromatograms with asymmetrical signals/insufficient chromatographic separation. Lyophilized material solved this problem. We noticed that protection from light, heat, and oxygen during extraction was essential for reproducible results so we took several measures to do so:
1. All extractions were performed in a dark room (only minimal indirect light) at 4 °C.
2. 0.1 w% BHT were added to the extraction solvents.
3. Extractions were performed swiftly and samples were analyzed directly.
4. Any homogenization of plant material was performed in liquid nitrogen under light shielded conditions.

We started method optimization of separation with an RP-C_{18} column but could not separate lutein and zeaxanthin properly. So, we switched to an RP-C_{30} column where we finally managed after some gradient optimization to separate all carotenoid isomers. The gradient described in the method section of the main publication was based on Gupta et al 2015 but needed to be fine-tuned to reproducibly separate all pigments in diverse lineages and species.

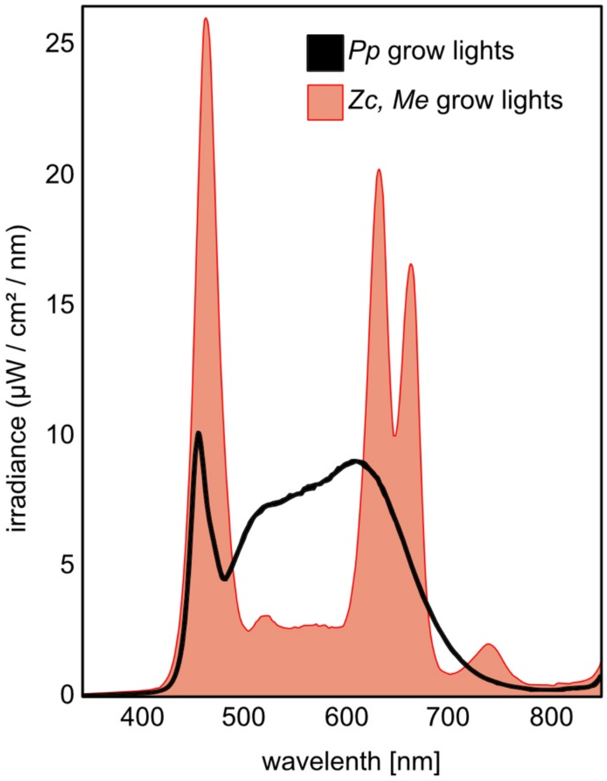

Supplementary Method Figure 1: Spectrum of growth lights used for the cultivation of *Physcomitrium patens* (*Pp*), *Zygnema circumcarinatum* (*Zc*), and *Mesotaenium endlicherianum* (*Me*).

Representative HPLC chromatogram of *Mesotaenium endlicherianium* and respective absorption spectra

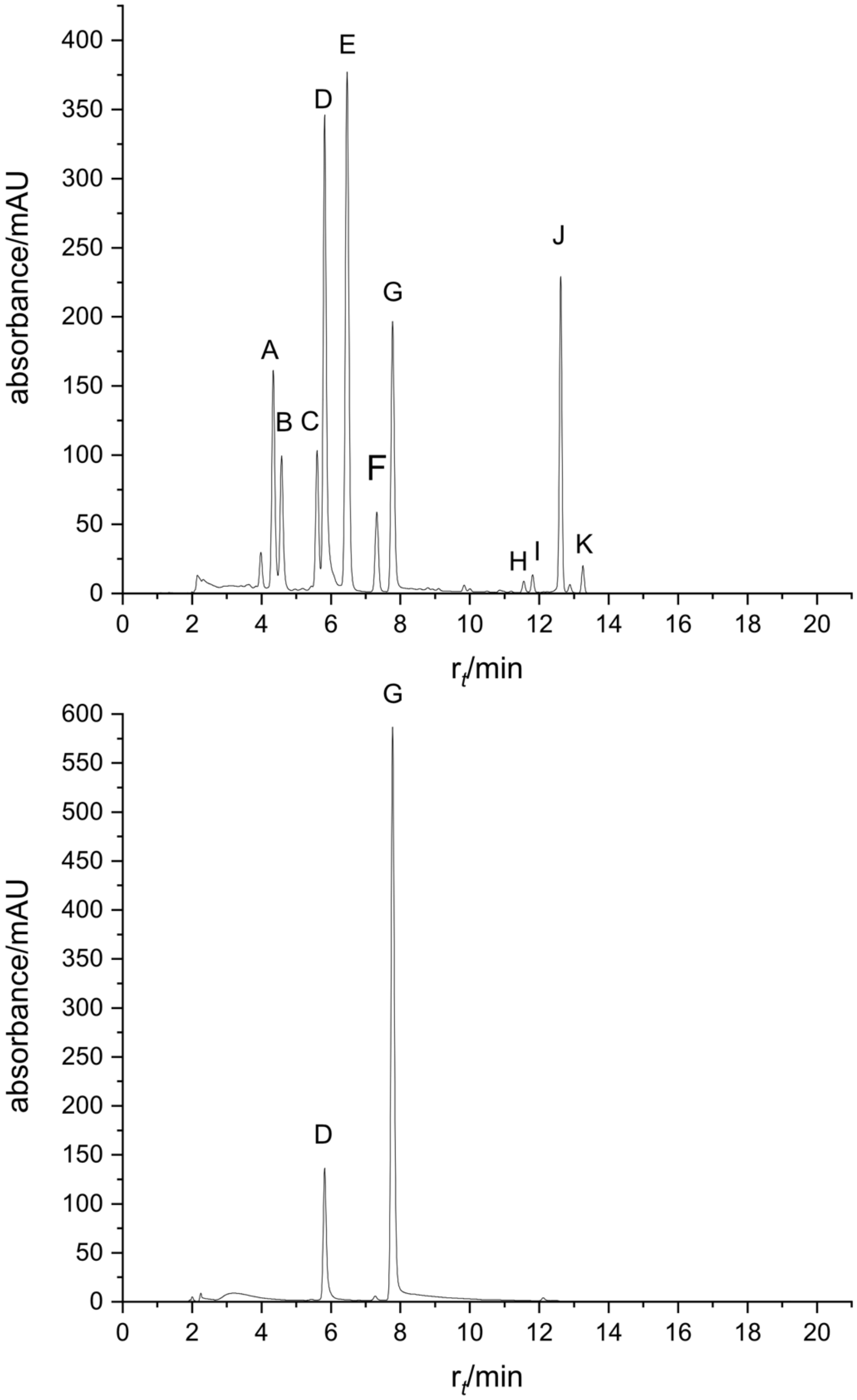

Supplementary Method Figure 2: HPLC chromatograms of *Mesotaenium endlicherianum* control replicate 1 recorded at 451 nm (top) and 660 nm (bottom). Violaxanthin (A), 9-*cis*-neoxanthin (B), antheraxanthin (C), chlorophyll b (D), lutein (E), zeaxanthin (F), chlorophyll a (G), 15-*cis*-β-carotene (H), α-carotene (I), β-carotene (J), 9-*cis*-β-carotene (K).

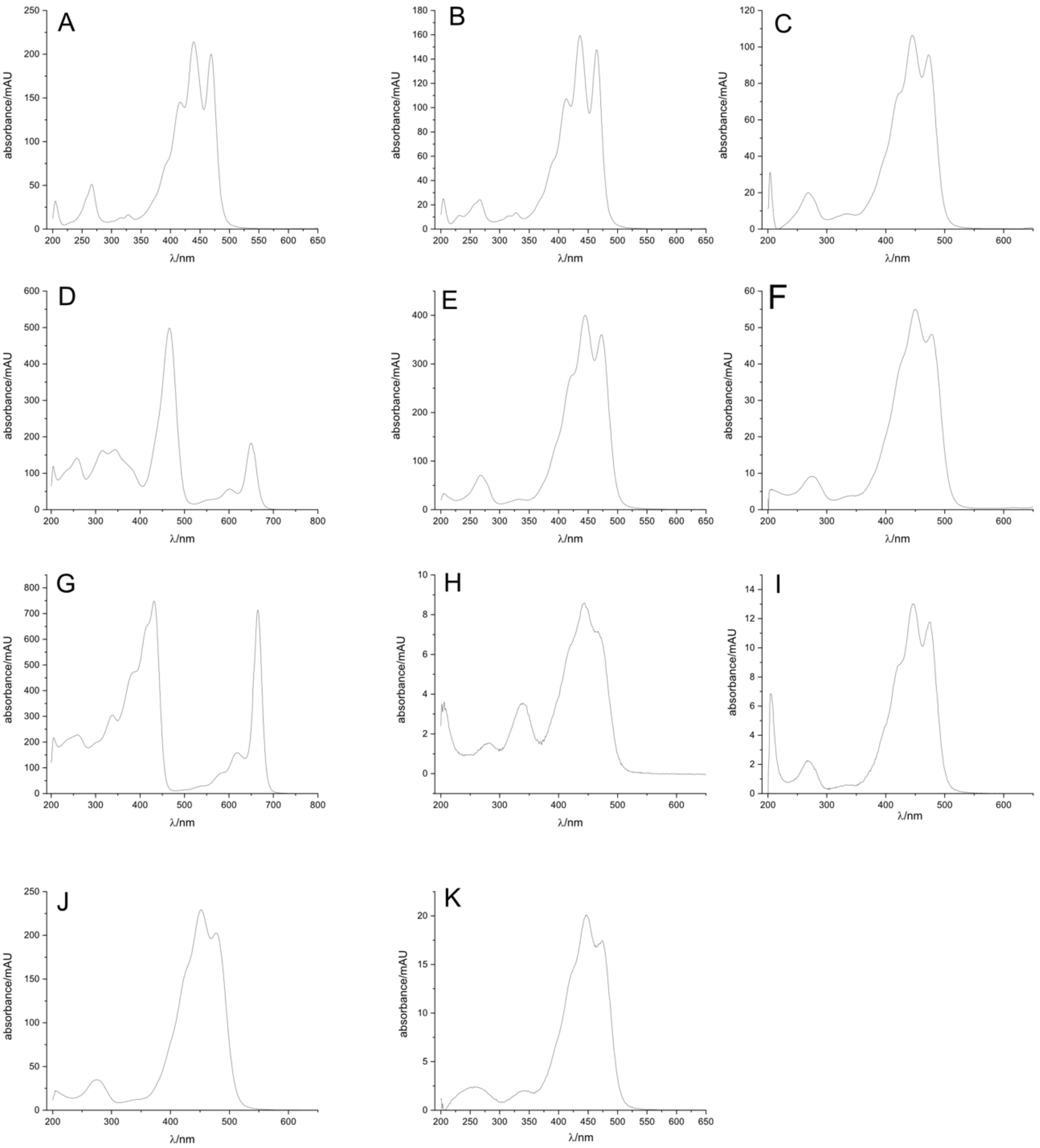

Supplementary Method Figure 3: Absorption spectra extracted from *Mesotaenium endlicherianum* control replicate 1. Violaxanthin (A), 9-*cis*-neoxanthin (B), antheraxanthin (C), chlorophyll b (D), lutein (E), zeaxanthin (F), chlorophyll a (G), 15-*cis*-β-carotene (H), α-carotene (I), β-carotene (J), 9-*cis*-β-carotene (K).

Supplementary Method Table 1: Calibration curves of carotenoid and chlorophyll standards

Pigment (retention time/min)	Effective amounts of calibration applied to column/µg	Linear regression (y = µg; x = mAU)	Detected absorption maxima (reported maxima [Gupta et al 2015])/ nm
violaxanthin (4.38)	0.0257, 0.257, 1.286, 2.571	y = $6.021 \cdot 10^{-4}$x	266, 416.5, 439, 469 (416, 439, 469)
9-*cis*-neoxanthin (4.58)	see violaxanthin	see violaxanthin and text below	266, 328, 413, 436, 465 (415.0, 437.0, 465.0)
antheraxanthin (5.60)	see zeaxanthin	see zeaxanthin and text below	268, (423), 445.5, 473.5 (424, 446, 474)
chlorophyll b (Milenković et al 2012) (5.82)	0.0271, 0.271, 1.357, 2.714	y = $5.897 \cdot 10^{-4}$x	344, 466, 601, 650 (461, 598, 648)
lutein (6.46)	0.0286, 0.286, 1.429, 2.857	y = $5.949 \cdot 10^{-4}$x	268, (423), 445, 473 (424, 445, 474)
zeaxanthin (7.32)	0.0257, 0.257, 1.286, 2.571	y = $2.646 \cdot 10^{-4}$x	276, (428), 450.5, 478 ((428.0), 451, 478)
chlorophyll a (Milenković et al 2012) (7.78)	0.0271, 0.271, 1.357, 2.714	y = $2.577 \cdot 10^{-4}$x	338, (383), (413), 432, (533), (581), 619, 665 (411, 431, 532, 581, 617, 662.5)
15-*cis*-β-carotene (11.55)	see β-carotene	see β-carotene	339, (424), 444, 467 (338.0, (420.0), 444.0, 468.0)
α-carotene (11.81)	0.0271, 0.271, 1.357, 2.714	y = $3.719 \cdot 10^{-4}$x	266, (420), 445.5, 475 (424, 446, 475)
β-carotene (12.62)	0.0266, 0.266, 1.329, 2.657	y = 0.00141x	275, (426), 452, 478 ((425.0), 452, 479)
9-*cis*-β-carotene (13.26)	see β-carotene	see β-carotene	256, 340, (421), 446, 473.5 ((420.0), 447.0, 473.0)
lycopene (19.42)	0.00643, 0.0643, 0.321, 0.643	$8.739 \cdot 10^{-5}$x	295, 445, 472, 503 (446.0, 472.0, 503.0)

Supplementary Method Table 2: Quotients of extinction coefficients used for calibration

Pigment	Extinction coefficient based on Thrane et al 2015 / L g^{-1} cm^{-1}	Quotient of extinction coefficients (E_2/E_1)
9-*cis*-neoxanthin (E_2)	233	0.9173
violaxanthin (E_1)	254	
antheraxanthin (E_2)	235	0.9592
zeaxanthin (E_1)	245	

Retention times of analytical standards and plant samples differed slightly due to effects of the plant matrix (and so also between organisms). Retention times based on *Mesotaenium endlicherianum* control replicate 1 except lycopene (based on standard), which did not accumulate under our conditions. The pistons appeared to be subject to relatively high loads, so that the retention times changed over time and the pistons had to be replaced more frequently than usual. Quality of analytical standards of vendors was strongly varying although treated with care (stored at –80 °C, weighted for calibration in the dark, measurements started on the same day as stock preparation etc.). Some were not mono isomeric and in the case of 9-*cis*-neoxanthin standard quality was so low that violaxanthin was used for calibration respecting differences in reported extinction coefficients (see table above). Antheraxanthin was quantified based on zeaxanthin calibration (since no commercial standard was at hand at this time) respecting the differences in reported extinction coefficients (see table above).

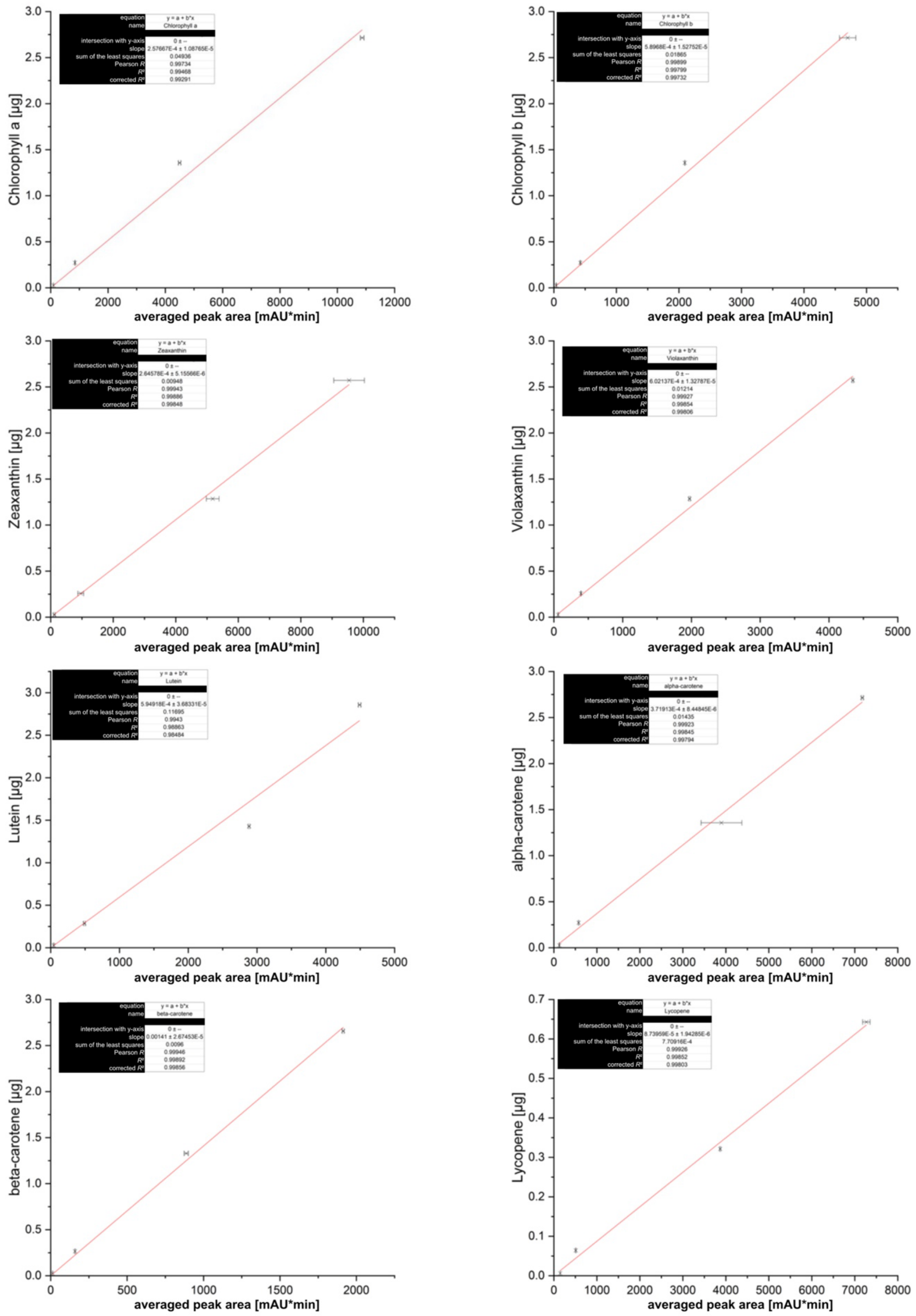

Supplementary Method Figure 4: Linear regressions of carotenoid and chlorophyll calibration data based on authentical analytical standard dilutions. X-axis: Averaged peak area of triplicate measurements in mAU*min; Y-axis: Respective metabolite and total amount of injected standard in µg.

HS-SPME-GC-MS

Recovery rate

HS-SPME methods do come with matrix effects during metabolite adsorption (and minor effects during desorption), either influenced by one or several metabolites or the complex matrices created by the biomass of the organisms. To normalize results the recovery rate of coinjected β-ionone-D_3 (4 µL) was applied. To determine this rate the measurements of the calibration (with β-ionone-D_3 coinjection) of 6-methyl-5-hepten-2-one (in total 9 replicates), the apocarotenoid standard with the lowest matrix effect on the β-ionone-D_3 signal (no significant effects) were used. The average signal of β-ionone-D_3 was used as reference ($R_{\beta\text{-ionone-D3}}$) for normalization and every signal detected was multiplied by the resulting recovery rate comparing the actual β-ionone-D_3 signal of each measurement with $R_{\beta\text{-ionone-D3}}$

$$\text{recovery rate} = \frac{R_{\beta-\text{ionone}-\text{D3}}}{\text{signal } \beta-\text{ionone}-\text{D3}}$$

Supplementary Method Table 3: Calibration curves of apocarotenoid standards

Apocarotenoid (m/z: **quantifying ion**, second qualifier, retention time)	Effective amounts of calibration applied to column/ng	Linear regression (y = ng; x = AU)
6-methyl-5-hepten-2-one (**108**, 126, 6.12)	24, 120, 240	$y = 7.107 \cdot 10^{-6}x$
β-cyclocitral (**137**, 152, 12.32)	0.522, 1.044, 5.22, 26.1, 52.2	$y = 1.562 \cdot 10^{-6}x$
β-ionone (**177**, 192, 19.20)	0.101, 0.202, 1.012	$y = 1.241 \cdot 10^{-8}x$
dihydroactinidiolide (**111**, 180, 20.19)	0.152, 0.304, 1.522, 7.612, 15.224	$y = 4.087 \cdot 10^{-7}x$

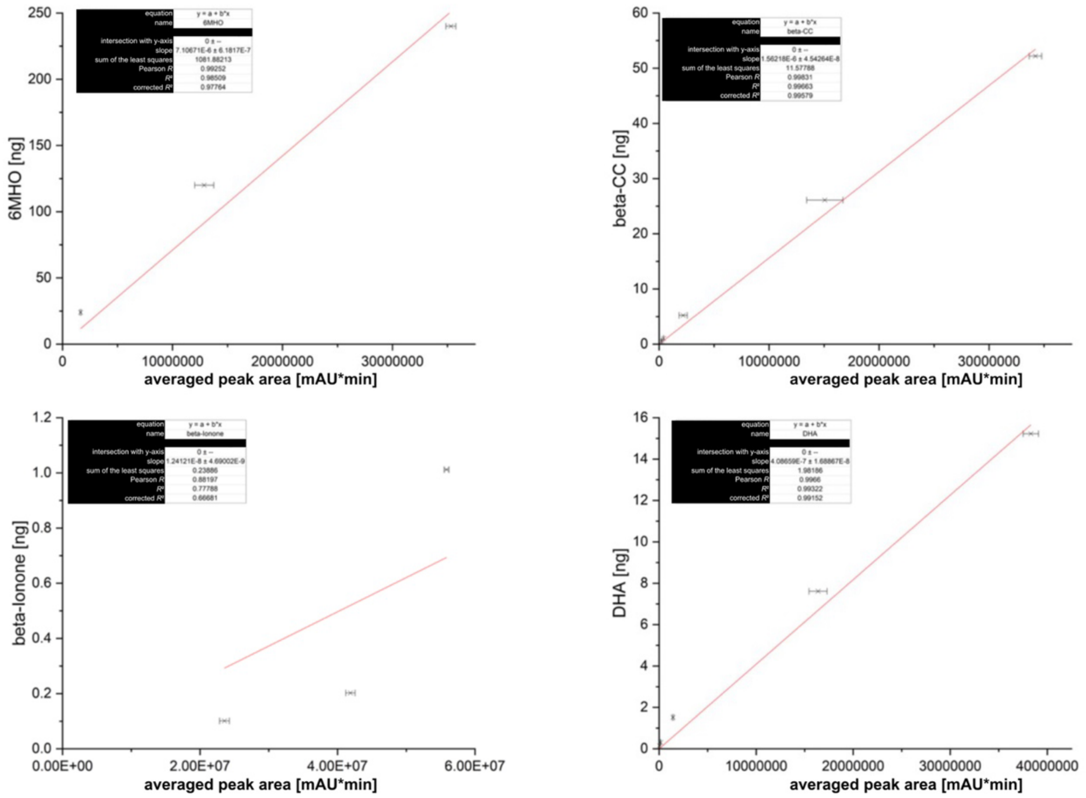

Supplementary Method Figure 5: Linear regressions of apocarotenoid calibration data based on authentical analytical standard dilutions. X-axis: Averaged peak area of triplicate measurements in mAU*min; Y-axis: Respective metabolite and total amount of injected standard in ng.

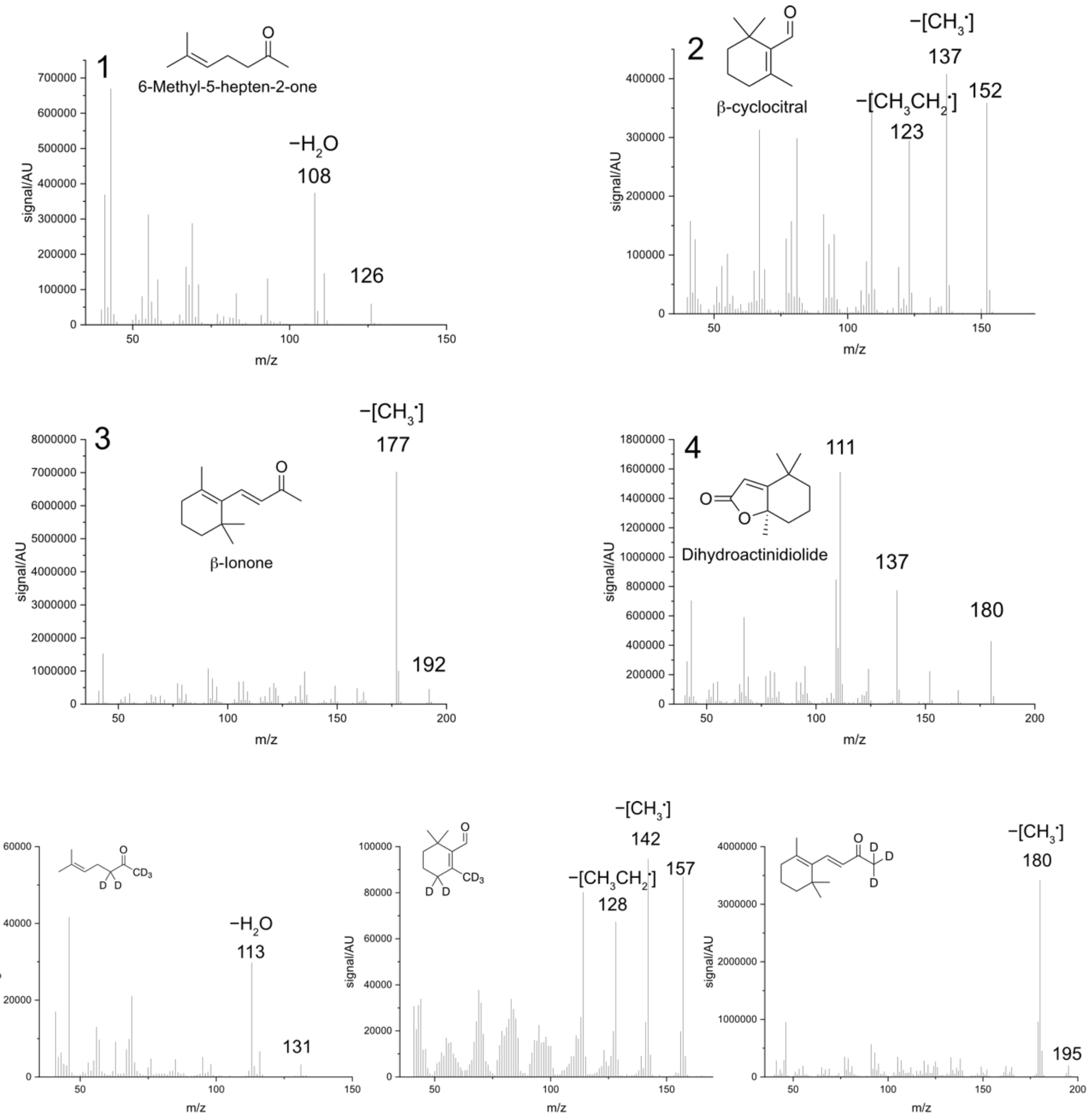

Supplementary Method Figure 6: Fragmentation patterns of authentical commercial standards and deuterated standards. Top: authentical commercial standards. Spectrum 1 shows the fragmentation pattern of 6MHO, with the molecular mass of 126 and the fragment obtained after loss of water (108). Spectrum 2 shows the fragmentation pattern of beta-cyclocitral with a molecular mass of 152, a fragment after loss of a methyl group (137) and a fragment that has lost an ethyl / two methyl groups (123). Spectrum 3 shows the fragmentation pattern of beta-ionone with a molecular mass of 192, and a fragment that has lost a methyl group (177). Spectrum 4 shows the fragmentation pattern of DHA with a molecular mass of 180, and two characteristic fragments of 137 and 111. Qualifying ions were picked based on literature, including Ramel et al. (2012) and Rivers et al. (2019). Bottom: deuterated standards.

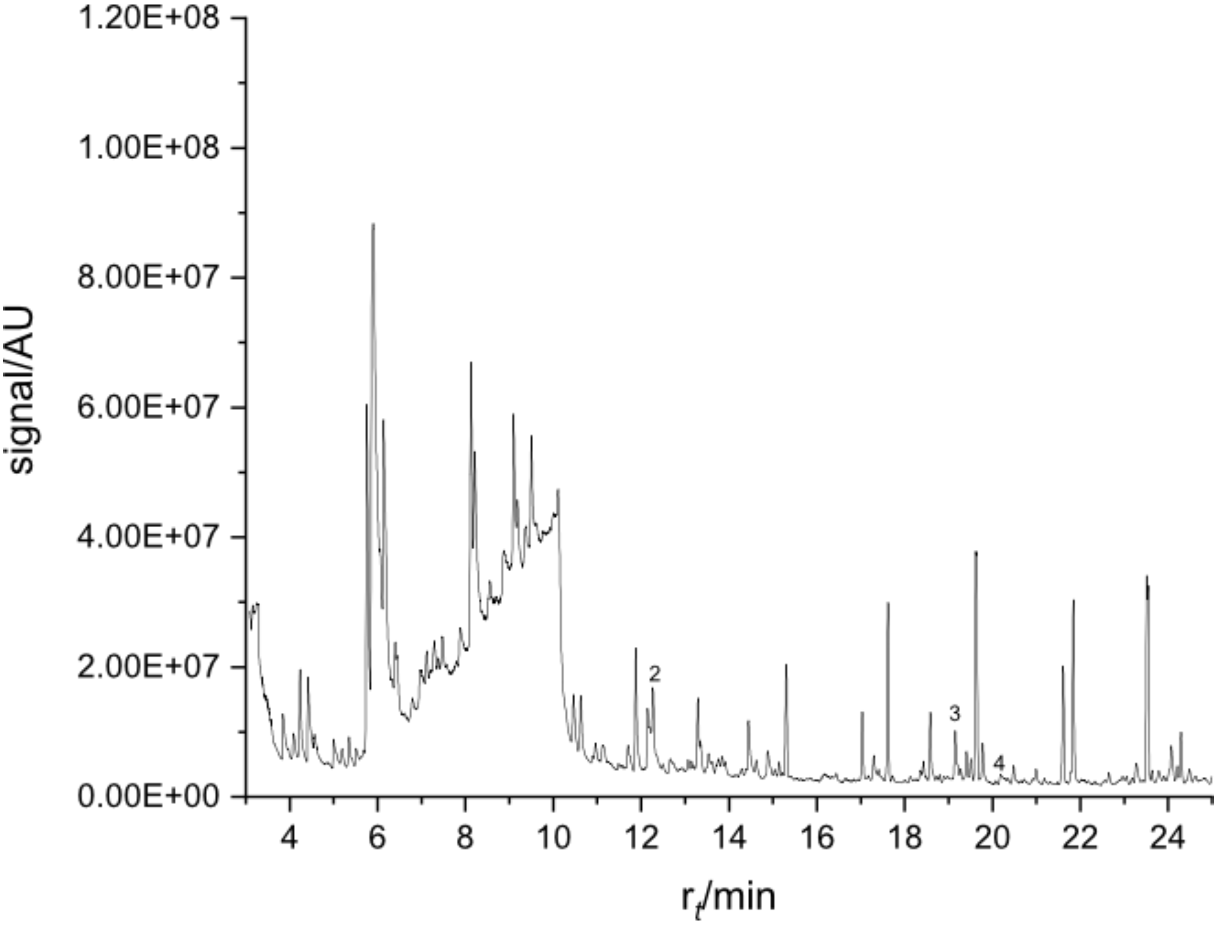

Supplementary Method Figure 7: Total ion chromatogram (TIC) of *Physcomitrium patens*. Homogenized flash frozen and lyophilized plant material was heated and volatiles were absorbed at the SPME fiber prior to injection and desorption. Detection was carried out using the scan mode of the mass spectrometer over a measuring time of 34.4 minutes; all relevant signals salient to apocarotenoids eluted before 24 minutes.

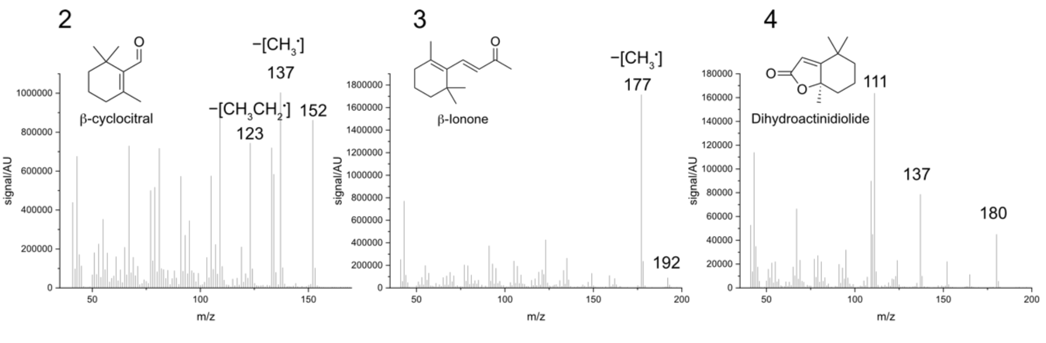

Supplementary Method Figure 8: Fragmentation pattern of apocarotenoids in *Physcomitrium patens*. Detection was based on informative fragmentation patterns as described for the commercial standards (see Supplementary Fig. 6).

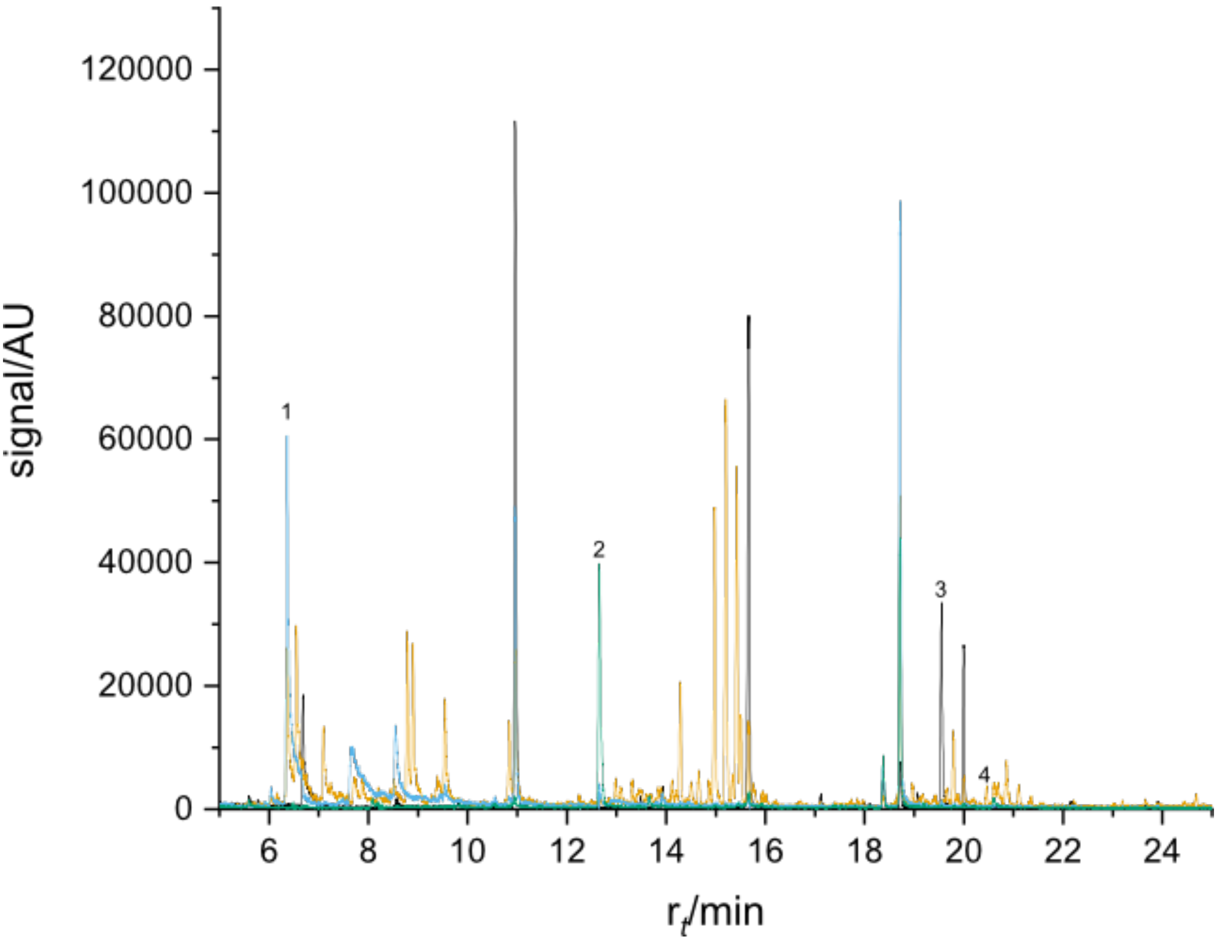

Supplementary Method Figure 9: Selected and merged ion chromatograms (XIC) of *Mesotaenium endlicherianum*. Measurements were performed in scan mode yielding TICs and later, during data analysis, selected ion chromatogram were merged to yield XICs. Homogenized flash frozen and lyophilized plant material was heated and volatiles were absorbed at the SPME fiber prior to injection and desorption. Detection was carried out using the scan mode of the mass spectrometer over a measuring time of 34.4 minutes; all relevant signals salient to apocarotenoids eluted before 24 minutes.

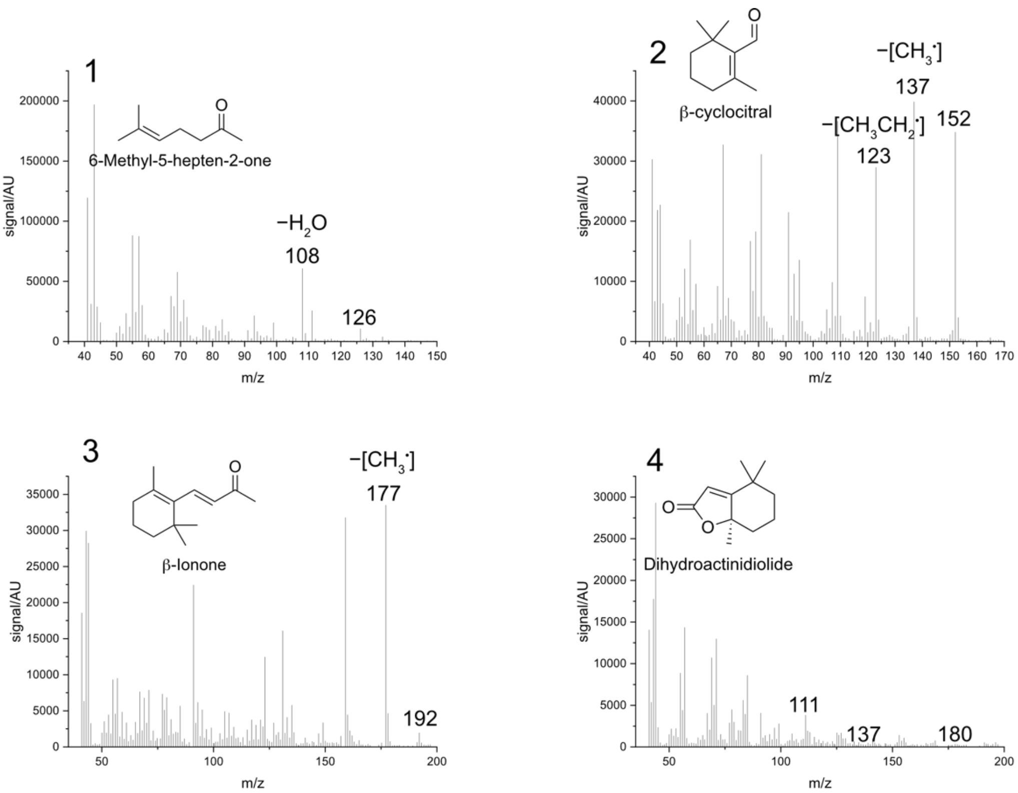

Supplementary Method Figure 10: Fragmentation pattern of apocarotenoids in *Mesotaenium endlicherianum*. Detection was based on informative fragmentation patterns as described for the commercial standards (see Supplementary Fig. 6).

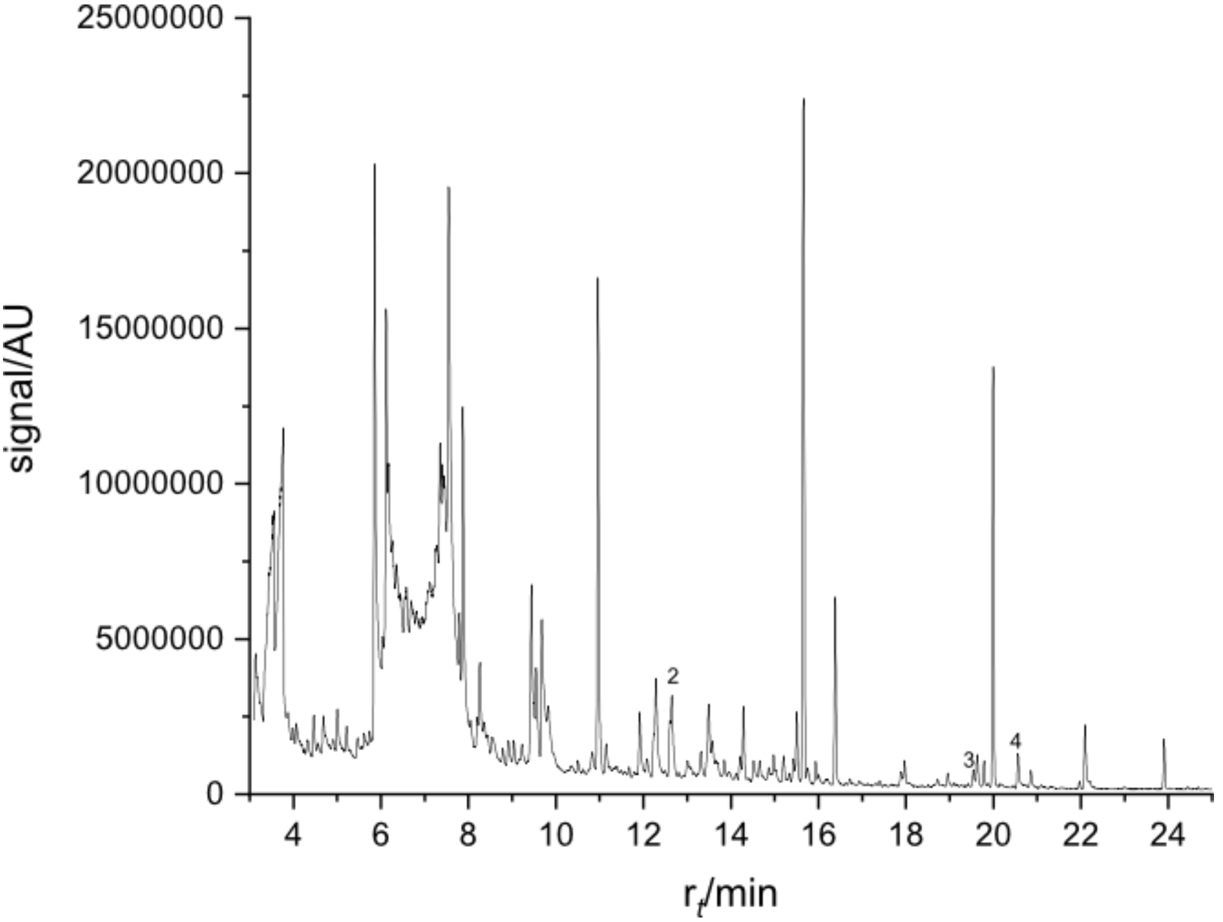

Supplementary Method Figure 11: Total ion chromatograms (TIC) of *Zygnema circumcarinatum*. Homogenized flash frozen and lyophilized plant material was heated and volatiles were absorbed at the SPME fiber prior to injection and desorption. Detection was carried out using the scan mode of the mass spectrometer over a measuring time of 34.4 minutes; all relevant signals salient to apocarotenoids eluted before 24 minutes.

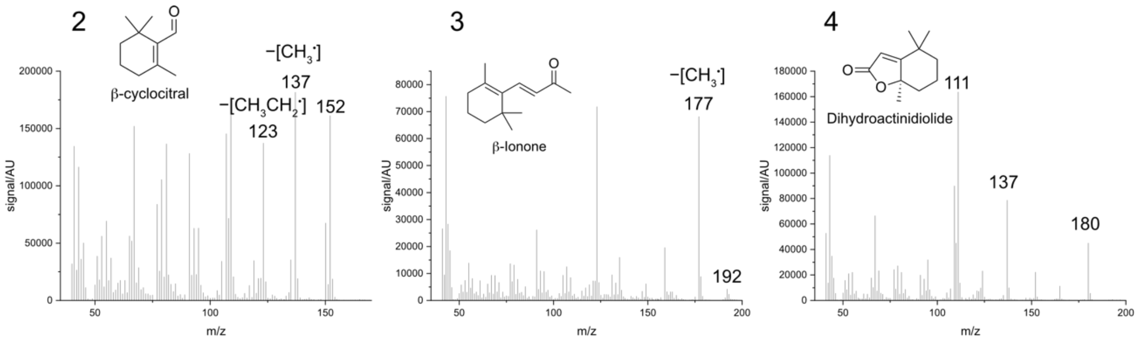

Supplementary Method Figure 12: Fragmentation pattern of apocarotenoids in *Zygnema circumcarinatum*. Detection was based on informative fragmentation patterns as described for the commercial standards (see Supplementary Fig. 6).

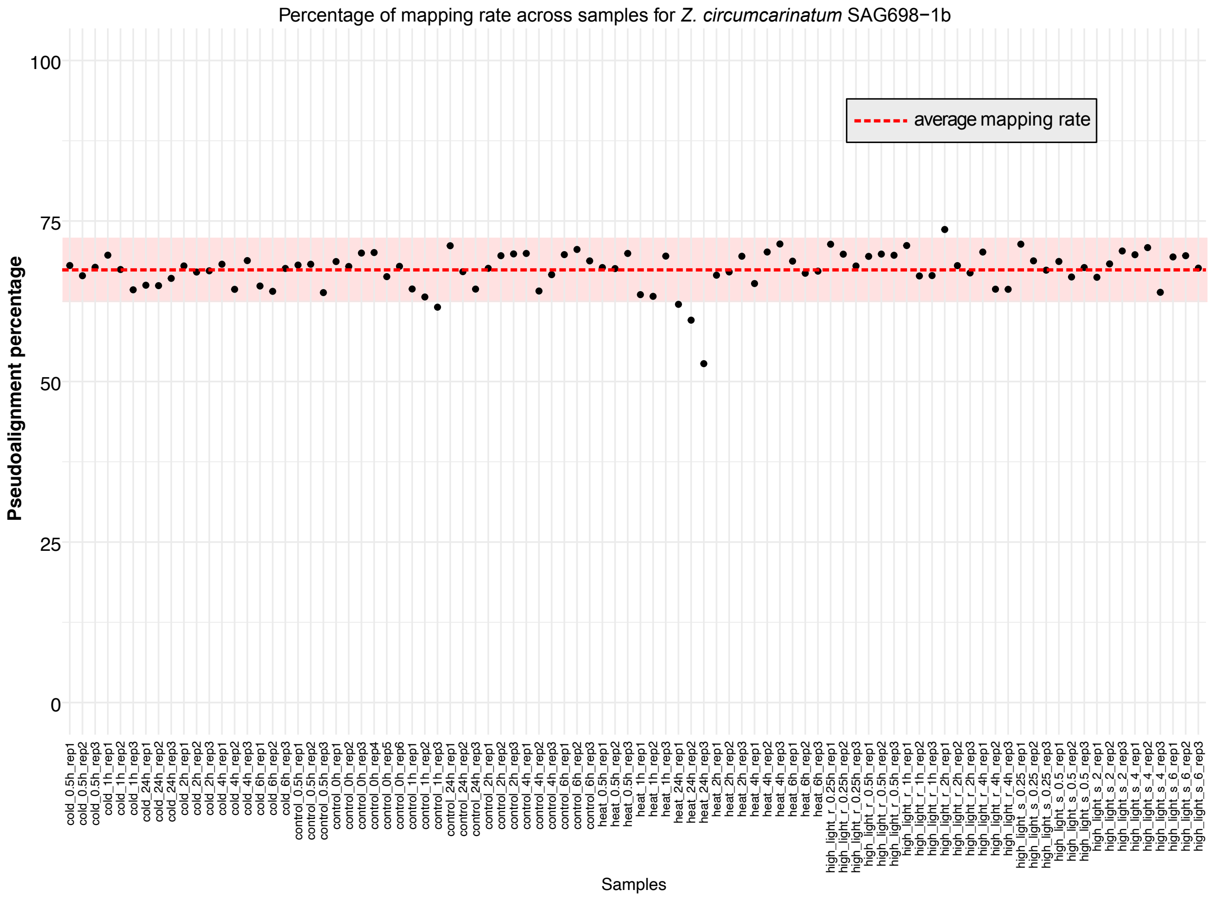

Supplementary Method Figure 13: Mapping rate, based on the pseudoalignment method kallisto, for the RNAseq samples garnered for *Zygnema circumcarinatum* SAG 698-1b. The average mapping rate is indicated by the red dotted line. Note that 85 out of 90 samples are within the ±5% range of the average mapping rate.

Extended rationale for co-expression analyses

We employed three major methods for network-based analyses of the time-course data in order to discern biological progams: WGCNA, DPGP, and SWING (Suppl. Method Figure 14). For diverse RNA-Seq analyses including WGCNA, DPGP, and SWING, it is essential to initially filter out genes exhibiting consistently low expression across samples. Furthermore, appropriate normalization and adjustment for non-biological parameters must be conducted.

WGCNA

This method involves initially computing the pairwise correlation between gene pairs across all samples utilizing techniques such as Pearson, Spearman, or biweight midcorrelation. Subsequently, to amplify robust correlations and diminish weak ones, we ought to identify a β (soft-threshold) through a grid search. The optimal β value will be selected to enable the network to approximate a scale-free topology. This is accomplished by employing a regression for the scale-free fit function and analyzing the mean and median number of connections within the network. The chosen soft-threshold will subsequently be employed to compute the adjacency matrix as follows:

$$a_{ij} = s_{ij}^{\beta}$$

s denotes the pairwise correlation between gene i and gene j. The subsequent step involves calculating the Topological Overlap Matrix (TOM) to assess the direct and indirect connections between gene pairs as outlined below:

$$TOM_{ij} = \frac{\sum_u a_{iu}a_{ju} + a_{ij}}{\min(k_i, k_j) + 1 - a_{ij}}$$

Where u represents all the genes in the network that are common neighbors between genes i and j. k_i denotes the connectivity (sum of adjacency weights) of gene i. The objective of calculating TOM is to identify robust biological connections by locating genes that share numerous neighbors rather than solely direct associations. Subsequently, we can compute the dissimilarity matrix utilizing TOM as follows:

$$D_{ij} = 1 - TOM_{ij}$$

We will utilize the dissimilarity matrix to cluster genes into distinct modules through hierarchical clustering, dynamically severing a distance tree at a designated height and combining similar clusters exhibiting high correlation. The user must select the soft-threshold, tree-cut level, and merging threshold according to recommended protocols, as each can influence the final clustering outcome. Users can identify interesting modules (clusters) by correlating the module eigengene (ME)—the primary principal component that encapsulates expression within the module—with external sample traits such as temperature and light intensity. An alternative method to identify intriguing modules is to compute Gene Significance (GS) to assess the strength of the correlation between each gene's expression and external traits. By computing the mean of gene significance (GS) for all genes within a module, we can also attribute GS to a module for each trait. Finally, enrichment analysis, such as Gene Ontology (GO) enrichment, can identify clusters of genes that are enriched with particular GO terms.

DPGP

Unlike many clustering techniques, such as hierarchical clustering or K-means clustering, this tool ascertains the number of clusters through a Dirichlet process (DP), which is entirely data-driven and conceptualized by resolving a Chinese restaurant process. The input must consist of gene expression profiles derived from time-series data. The Dirichlet Process, a Bayesian non-parametric model, estimates the number of clusters without prior specification. This method presupposes a "rich-get-richer" clustering behavior, indicating that larger clusters are more inclined toward expansion.

Upon establishing the number of clusters, genes are allocated to clusters based on the premise that genes within each cluster adhere to a Gaussian Process (GP). The Gaussian Process models temporal dependencies within clusters and disseminates a mean and covariance structure for the trajectories. DPGP employs a squared exponential kernel to assess similarity between time points as follows:

$$\kappa(xt, xt') = \tau^2 exp\left(-\frac{||xt - xt'||^2}{2l^2}\right)$$

Where κ denotes the Kernel and τ^2 signifies the amplitude of variations and l represents the smoothness of trajectories. The algorithm employs an iterative Gibbs sampling method. During the initialization phase, each gene is allocated to an individual cluster. Gaussian Process parameters (mean, variance, etc.) are initialized using default or prior values. For each gene, the probability of assignment to each cluster is computed using Markov Chain Monte Carlo (MCMC), based on Likelihood (the gene's fit to the cluster's Gaussian Process model) and Prior (the cluster size and the concentration parameter of the Dirichlet process).
Each gene is subsequently allocated to the most likely cluster or a new cluster if warranted. Parameters specific to the cluster (mean, variance, etc.) are subsequently revised. The parameters of the covariance kernel (l and τ^2) are optimized through the marginal likelihood. The average trajectory is revised as follows:

$$\mu_h = K(x, x)[K(x, x) + \sigma^2 I]^{-1} y_h$$

K(x,x) denotes the covariance matrix for cluster h, σ^2 represents the marginal variance, and y_h signifies the data for cluster h. The aforementioned steps are reiterated across MCMC iterations (default: n=1000) to sample from posterior distributions. The algorithm generates the Maximum a Posteriori (MAP) Partition, representing the most probable clustering configuration, and the Posterior Similarity Matrix (PSM), which indicates the likelihood that pairs of genes belong to the same cluster. We identified interesting clusters of tightly regulated genes by selecting those assigned to a cluster with a probability of no less than 0.7. Subsequently, we conducted Gene Ontology enrichment analysis to determine if any biological processes are enriched within these tightly regulated gene clusters.

SWING

The idea behind this tool is to compare time-series prediction models to infer Granger causality. It uses only and only time-series gene expression profile, and receives the size of the window (of time points) to calculate Granger causality via a windowed strategy over time. During the modeling, each gene will be treated as a response variable, while other genes (at different time delays) serve as explanatory variables. Here, user should define the minimum and maximum time delays that are meaningful based on the biological question at hand and experiment design. Then, using a

Random Forest algorithm determines feature importance scores for each window of time using a multivariate Granger causality (via calculating the mean squared error). An adjacency matrix will be created for each window where a_{ij} is the inferred score for gene i regulating gene j. The rank of an edge in each windowed model can be used as the confidence metric to compare across methods. SWING does two aggregations. Confidence values from windowed subsets are aggregated into a single network by taking the mean rank of the edge at each delay k, and then taking the mean rank of the edge across all delays. The window specific models (aka adjacency metrices) are aggregated to create a consensus network by aggregating edge ranks across windows and delays and computing the mean and median for each edge. The output will be a directed edge list (aka graph) that shows regulatory relationship predicted via this method and their confidence scores.

Multivariate Granger causality evaluates whether past values of a gene improve the prediction of another gene. It also incorporates multiple explanatory genes and time delays. We used the Random forest option with 500 ensemble of decision trees to assess Granger causality in time-series gene expression data. Permutation testing is also used to establish null distribution for feature importance scores. By randomly shuffling explanatory variables, SWING can evaluate whether observed scores significantly exceed what is expected by chance or not. The edges that are not consistently ranked highly across multiple windows are removed during the aggregation step.

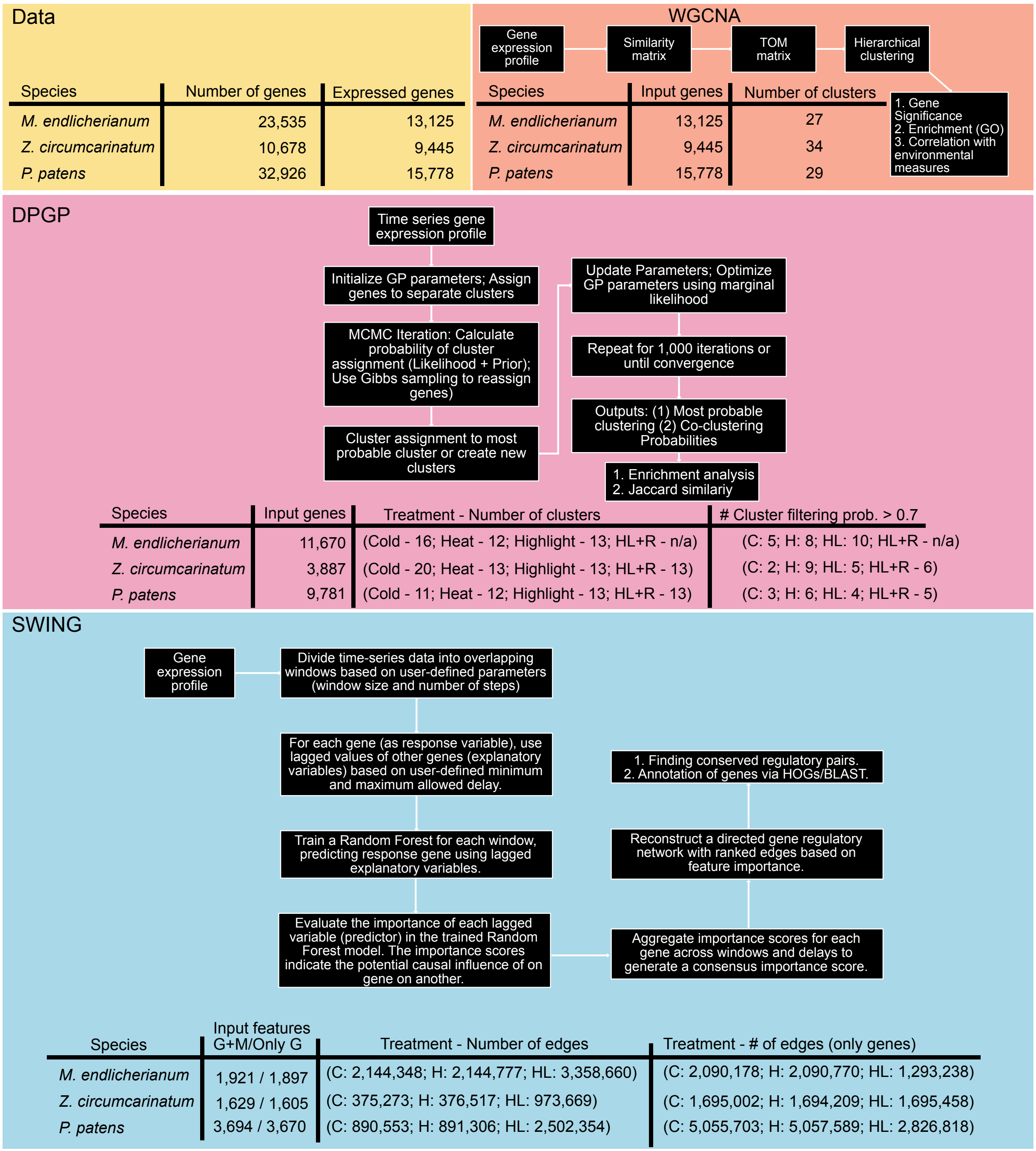

Supplementary Method Figure 14: Workflows employed for the network analyses.
