# Supplementary material for: Time-resolved oxidative signal convergence across the algae–embryophyte divide
Source: Nat Commun. 2025 Feb 19;16:1780. doi: 10.1038/s41467-025-56939-y (PMC11840003; doi:10.1038/s41467-025-56939-y)
Supplement: Supplementary file 2 — Reporting Summary [file 41467_2025_56939_MOESM2_ESM.pdf]

nature portfolio

Corresponding author(s): Jan de Vries

Last updated by author(s): Jan 22, 2025

Reporting Summary

Nature Portfolio wishes to improve the reproducibility of the work that we publish. This form provides structure for consistency and transparency in reporting. For further information on Nature Portfolio policies, see our Editorial Policies and the Editorial Policy Checklist.

Statistics

For all statistical analyses, confirm that the following items are present in the figure legend, table legend, main text, or Methods section.

n/a	Confirmed	
☐	☒	The exact sample size (*n*) for each experimental group/condition, given as a discrete number and unit of measurement
☐	☒	A statement on whether measurements were taken from distinct samples or whether the same sample was measured repeatedly
☐	☒	The statistical test(s) used AND whether they are one- or two-sided<br>*Only common tests should be described solely by name; describe more complex techniques in the Methods section.*
☐	☒	A description of all covariates tested
☐	☒	A description of any assumptions or corrections, such as tests of normality and adjustment for multiple comparisons
☐	☒	A full description of the statistical parameters including central tendency (e.g. means) or other basic estimates (e.g. regression coefficient) AND variation (e.g. standard deviation) or associated estimates of uncertainty (e.g. confidence intervals)
☐	☒	For null hypothesis testing, the test statistic (e.g. *F*, *t*, *r*) with confidence intervals, effect sizes, degrees of freedom and *P* value noted<br>*Give P values as exact values whenever suitable.*
☒	☐	For Bayesian analysis, information on the choice of priors and Markov chain Monte Carlo settings
☒	☐	For hierarchical and complex designs, identification of the appropriate level for tests and full reporting of outcomes
☐	☒	Estimates of effect sizes (e.g. Cohen's *d*, Pearson's *r*), indicating how they were calculated

Our web collection on statistics for biologists contains articles on many of the points above.

Software and code

Policy information about availability of computer code

Data collection	Photophysiological measurements were carried out using a MINI PAM II (Waltz, Germany) and values were directly recorded. Sequencing data was collected on an Illumina NovaSeq6000 platform operated by Novogene UK. wget (GNU Wget 1.14) was used to download genomic sequences from Phytozome, Novogene, DDBJ, Figshare, Fernbase, and TAIR databases. A HPLC system Agilent 1100 series equipped with a UV-Vis-DAD detector was used for simultaneous carotenoid and chlorophyl measurements. For separation a YMC Carotenoid C30 S- 3 µm column (250 x 4.6 mm I.D.) from YMC Europe was integrated. HPLC method programming and data aquisition was performed with the software ChemStation for LC 3D systems (Rev. B.04.03 [16]; Agilent Technologies). The system used for volatile apocarotenoid measurements was comprised of the following technical compartments: GC/MSD instrument (Agilent Technologies 7890B) coupled to a 5977B MSD quadrupole, PAL3 Auto sampler system with Robotic Tool Change (RTC 120), polydimethylsiloxane/divinylbenzene/carboxen (50/30 µM DVB/CAR on PDMS) adsorbent SPME fiber from Supelco, HP-5MS UI column (30 m x 0,25 mm; 0,25 µm coating thickness; Agilent). GC-MS method promming was carried out using the "Enhanced Mode" of MassHunter Data Analysis (MassHunter GC/MS Acquisition B.07.05.2479 23-Aug-2016; Agilent Technologies).
Data analysis	We installed all packages (and their dependencies) using conda if possible. Otherwise, we followed the installation guide offered by the authors of the tool. For R packages, we installed them from, in this order of priorities, from Cran repository, BioCmanager, or the tool GitHub instructions. All codes used to perform computational analyses are available in our GitLab repository:<br>https://gitlab.gwdg.de/armin.dadras/time-resolved-oxidative-signal-convergence-across-the-algae-embryophyte-divide<br>Code is also available on Zenodo under doi: 10.5281/zenodo.14710622<br><br>Metabolite analysis of HPLC data was carried out using the software ChemStation for LC 3D systems (Rev. B.04.03 [16]; Agilent Technologies). For data obtained via GC-MS, analyses were carried out using Enhanced ChemStation (MSD ChemStation D.01.02.16 15JUNE-2004; Agilent Technologies).

Functional annotation
In order to assign functional information to the sequences we employed an comprehensive set of tools including InterProScan (Jones et al. 2014) (v5.64-96.0 and -pa -goterms flags), eggNOG-mapper (Buchfink et al. 2021; Cantalapiedra et al. 2021; Huerta-Cepas et al. 2019) (v2.1.12 and -m diamond--dmnd_iterate yes--dbmem --cpu 0 --evalue 1e-10 --sensmode ultra-sensitive --tax_scope 33090 --dmnd_db eggnog_proteins_default_viridiplantae.dmnd flags), BLAST (Camacho et al. 2009) (v2.15.0) against protein files of A. thaliana(Cheng et al. 2017) and genome scale gene family analysis using Orthofinder (Emms and Kelly 2015, 2019; Katoh and Standley 2013; Price et al. 2010) (v2.5.5). Me, Zc SAG698-1b and Pp had 74.5, 93.9, and 78.1% of their genes in HOGs and they have 8.2, 2.2, 12% or their genes in species-specific orthogroups, respectively. We first ran Orthofinder with these settings: -S diamond -M msa -A mafft -T fasttree -t 200 -a 6 -y. Building on this, we redid the analysis by providing a user-defined rooted species tree to increase the accuracy of the inference and this tree includes the following species: Anthoceros agrestis oxford (Li et al. 2020a), Azolla filiculoides (Li et al. 2018), A. thaliana (Cheng et al. 2017), Brachypodium distachyon (The International Brachypodium 2010), Chara braunii (Nishiyama et al. 2018), Chlorokybus melkonianii(Wang et al. 2020; Irisarri et al. 2021), Chlamydomonas reinhardtii (Merchant et al. 2007), Closterium sp. NIES-67(Sekimoto et al. 2023), Klebsormidium nitens (Hori et al. 2014), Mesotaenium endlicherianum (Cheng et al. 2019a; Dadras et al. 2023), Marchantia polymorpha (Montgomery et al. 2020), Mesostigma viride (Cheng et al. 2019b), Ostreococcus lucimarinus (Palenik et al. 2007), Oryza sativa (Ouyang et al. 2007), Prasinoderma coloniale (Li et al. 2020b), Penium margaritaceum (Jiao et al. 2020), Physcomitrium patens(Lang et al. 2018), Solanum lycopersicum (The Tomato Genome 2012), Selaginella moellendorffii (Banks et al. 2011), Spirogloea muscicola (Cheng et al. 2019a), Zygnema circumcarinatum (Feng et al. 2024) SAG 698-1a, Z. circumcarinatum(Feng et al. 2024) SAG 698-1b, Z. circumcarinatum (Feng et al. 2024) UTEX 1559, Z. circumcarinatum (Feng et al. 2024) UTEX 1560, and Zea mays (Jiao et al. 2017). To assign GO terms to each gene, we combined the functional annotation of InterProScan and eggNOG-mapper into a table for each species using the ontologyIndex package (v2.11) (Greene et al. 2017). We also used Tapscan (v2)(Wilhelmsson et al. 2017) to identify transcription factors for each species.

Quality control and gene expression quantification and exploratory data analysis
We used the pipeline of (Dadras et al. 2023) built using Snakemake (v7.7.0) (Mölder et al. 2021) and available on GitHub (https://github.com/dadrasarmin/rnaseq_quantification_kallisto_pipeline). Briefly, we used FastQC (v0.12.1) (Andrews 2010) and MultiQC (v1.16)(Ewels et al. 2016) to perform quality control, Trimmomatic (v0.39) (Bolger et al. 2014) to perform trimming and filtering, and Kallisto (v0.48.0)(Bray et al. 2016) to quantify gene expressions.
We used R (v4.3.2) (R Core Team and Team 2022) and tidyverse (v2.0.0) (Wickham et al. 2019) for data analysis and visualization. We used tximport (v1.30.0) (Soneson et al. 2015) to import and summarize count tables at gene-level into R and normalized count tables for both sequencing depth and gene length using the following settings: “countsFromAbundance = "lengthScaledTPM", txOut = F”. We used edgeR (v4.0.6) (Robinson et al. 2010) to keep only genes with expression levels higher than 10 counts-per-milion (CPM) in at least 3 samples. Based on the experimental design of this study, we chose to perform global normalization (quantile normalization) to remove technical unwanted variations in our dataset (Hicks and Irizarry 2015). We used qsmooth (v1.18.0) (Hicks et al. 2018) with treatments as group_factor to perform the normalization.

Differential gene expression analysis
We used limma (v3.58.1) (Ritchie et al. 2015) to model gene expression changes under each treatment samples compared to the same time point in the control condition using lmFit, contrasts.fit, eBayes, decideTests functions. We picked |log2(fold change)| >= 1 as well as Benjamini-Hochberg method for p-value adjustment and a threshold of 0.05 to determine differentially expressed genes (DEGs). We used GO-gene tables that we prepared in the functional annotation step to perform Over-Representation Analysis (ORA) using clusterProfiler (v4.10.0) (Wu et al. 2021). In this section, we only focused on “Biological Process” domain of GO terms, using only expressed genes in our dataset as background, adjusted p. value cut off <= 0.05 and q. value cut off <= 0.05 for enrichment analysis. To visualize the general pattern of GO term enrichment over-time under each treatment, we used alluvial (v0.2.0)(Brunson 2020) and picked top 10 GO terms that are enriched in as many as possible time points and sorted them on Y-axis based on the enriched gene count of the GO term. The thickness of each stratum is visualized based on the number of enriched genes in each GO term.

Co-expression network analysis
It is well known that gene co-expression methods, each with its own strengths and weaknesses, can lead the different final networks (Chowdhury et al. 2019). In this study, we used two methods from different classes of co-expression network analysis. First, we use Weighted Gene Co-expression Network Analysis (WGCNA v1.72.5) (Langfelder and Horvath 2008) to infer one network from all treatments and time points per species. In this method, correlation measures are used to calculate an adjacency matrix using a beta and a network type. Next, the topological overlap matrix is calculated based on the adjacency matrix, then a distance matrix will be calculated and using hierarchical clustering genes will be divided into various modules. Finally, modules that are very similar based on their Eigenvalues will be merged. We followed the authors’ recommendations for the parameters for this last step. In summary, we screened soft-thresholding powers from 1 to 50 for each species and picked a soft threshold based on mean connectivity (around 50), median connectivity (around 20), and signed R2 of Scale free topology model fit (above 0.8). We picked 20, 20, 14 as soft threshold for Me, Pp, and Zc. We built our networks using the following settings: Merging threshold=0.20, correlation method= biweight midcorrelation, network type=signed, TOMType=signed, minimum module size=30, and maximum percentile of outliers=0.05. We calculated Pearson’s correlation coefficient and gene significance based on module’s Eigengene values and various physiological measurements and metabolite concentration changes. We also calculated inter- and intra-modular connectivity for each module and picked top 20 highly connected genes as the hubs of that module. For each module, we performed GO enrichment analysis similar to the differential gene expression analysis mentioned above. Biological theme comparison plots were made using clusterProfiler to discover patterns of GO enrichment among different modules. We used igraph (v1.6.0) (Csardi and Nepusz 2006) to visualize co-expression network for each module and annotate the hubs. We annotated hubs based on the blast results described in the functional annotation above in this order: (a) gene symbol > (b) A. thaliana best hit gene ID > (c) species gene ID.
The second method is the Dirichlet Process Gaussian Process mixture model (DPGP), a non-parametric model-based method that is designed to perform gene co-expression analysis for time series datasets. It solves the problem of the number of clusters using a Dirichlet process and then model the dependencies in gene expression profiles between time points using a Gaussian model (McDowell et al. 2018). We used fold change values that has a significant adjusted p-value ≤ 0.05 compared to the same time point in control as the input of the software. Due to assumptions of this method, we had to make one network per species (Me, Zc, Pp) and per treatment (cold, heat, HL); nine networks in total. We visualized expression profiles and performed GO enrichment analysis as mentioned above. The authors of DPGP suggested that this tool can be used to look for tightly regulated genes by filtering for gene assignments to clusters with a specific threshold in the final probability. We picked probability ≥ 0.7 as our threshold as suggest by the DPGP authors and compared inter- and intra-species similarities between clusters using Jaccard distance.
We picked a collection of the most similar filtered gene clusters based on Jaccard distances to investigate further. We normalized the data between 0 to 1 to visualize it as a heatmap. Also, we put a minimum cap of 0.9 Jaccard distance for both clustering methods since the heatmap was not informative due to presence of few outliers in pair-wise combinations (very close clusters).

Gene regulatory network (GRN) inference
There are various methods to calculate GRN based on time series transcriptomics but the balance between run time and accuracy makes it hard to pick a gold standard among all methods. Here, we used Sliding Window Inference for Network Generation (SWING) (Finkle et al. 2018) to account for our temporal information which is one of the best method for this purpose according to independent benchmarkings (Lu et al. 2021). SWING uses a multivariate Granger causality to infer network topology from time series data. We combined the transcriptomics data with metabolite concentrations as inputs and used the Random Forest (RF, i.e. SWING-RF) method to infer the network which has the best performance compared to LASSO and PLSR in the benchmarking (Lu et al. 2021). The parameters that should be defined to infer the network were decided based on the best practice that was suggested by the authors of SWING as follows; For Me, we had more metabolite data and we picked: minimum lag= 0, maximum lag = 1, fixed-length of sliding window = 4 and number of trees = 500. For Pp and Zc, we picked these parameters: minimum lag = 0, maximum lag = 1, fixed-length of sliding window = 2 and number of tress = 500. We performed Z-score transformation on the input datasets. To integrate scores from many windows and delays into a single score (regulator-regulated pairs), we utilized this package's mean-mean aggregation approach. Confidence values from windowed subsets are combined into a single network by calculating the mean rank of the edge at each delay k, followed by the average rank of the edge over all delays.
The outcome of this method is a ranked list of all possible pairs ordered from the most to the less confident one. We first filtered out pairs with 0 support, extracted the top 0.1%, and visualized the result via igraph. Since it was still a very big network, then investigated the network with more filtering. Basically we created file list based on keywords downloaded from TAIR. (a) Cold consists of “cold acclimation”, “response to cold”, and “cellular response to cold” (b) Heat consists of “response to heat”, “heat acclimation”, “cellular response to heat”, and “cellular heat acclimation” (c) HL consists of “response to high light intensity”, and “cellular response to high light intensity” (d) Oxidative consists of “response to oxidative stress”, “cellular response to oxidative stress”, “cellular response to reactive oxygen species”, “response to photooxidative stress”, and “regulation of response to oxidative stress” (e) “Carotenoid metabolic process” (f) “Apocarotenoid metabolic process”. We then used the BLAST results mentioned above to find possible homologs of these genes in our species of interest. We used these gene sets as well as metabolite list and TF list extracted using TapScan to look for top 0.1% edges of GRN for each of these subsets. We used igraph to visualize the data and annotate the top 100 nodes in the edge list as explained in the co-expression network section.

For manuscripts utilizing custom algorithms or software that are central to the research but not yet described in published literature, software must be made available to editors and reviewers. We strongly encourage code deposition in a community repository (e.g. GitHub). See the Nature Portfolio guidelines for submitting code & software for further information.

Data

Policy information about availability of data

All manuscripts must include a data availability statement. This statement should provide the following information, where applicable:

- Accession codes, unique identifiers, or web links for publicly available datasets
- A description of any restrictions on data availability
- For clinical datasets or third party data, please ensure that the statement adheres to our policy

All RNAseq reads have been uploaded to NCBI SRA and can be accessed under Bioproject PRJNA895341 (Mesotaenium) and PRJNA939006 (Zygnema and Physcomitrium) and SRA accessions SRR22077315 SRR22077315, SRR22077316, SRR22077317, SRR22077318, SRR22077319, SRR22077320, SRR22077321, SRR22077322, SRR22077323, SRR22077324, SRR22077325, SRR22077326, SRR22077327, SRR22077328, SRR22077329, SRR22077330, SRR22077331, SRR22077332, SRR22077333, SRR22077334, SRR22077335, SRR22077336, SRR22077337, SRR22077338, SRR22077339, SRR22077340, SRR22077341, SRR22077342, SRR22077343, SRR22077344, SRR22077345, SRR22077346, SRR22077347, SRR22077348, SRR22077349, SRR22077350, SRR22077351, SRR22077352, SRR22077353, SRR22077354, SRR22077355, SRR22077356, SRR22077357, SRR22077358, SRR22077359, SRR22077360, SRR22077361, SRR22077362, SRR22077363, SRR22077364, SRR22077365, SRR22077366, SRR22077367, SRR22077368, SRR22077369, SRR22077370, SRR22077371, SRR22077372, SRR22077373, SRR22077374, SRR22077375, SRR22077376, SRR22077377, SRR22077378, SRR22077379, SRR22077380, SRR22077381, SRR22077382, SRR22077383, SRR22077384, SRR22077385, SRR22077386, SRR22077387, SRR22077388, SRR22077389, SRR22077390, SRR22077391, SRR22077392, SRR22077393, SRR22077394, SRR22077395, SRR22077396, SRR22077397, SRR22077398, SRR22077399, SRR22077400, SRR22077401, SRR22077402, SRR22077403, SRR22077404, SRR22077405, SRR22077406, SRR22077407, SRR22077408 SRR22077409 (Mesotaenium); and SRR23625966, SRR23625967, SRR23625968, SRR23625969, SRR23625970, SRR23625971, SRR23625972, SRR23625973, SRR23625974, SRR23625975, SRR23625976, SRR23625977, SRR23625978, SRR23625979, SRR23625980, SRR23625981, SRR23625982, SRR23625983, SRR23625984, SRR23625985, SRR23625986, SRR23625987, SRR23625988, SRR23625989, SRR23625990, SRR23625991, SRR23625992, SRR23625993, SRR23625994, SRR23625995, SRR23625996, SRR23625997, SRR23625998, SRR23625999, SRR23626000, SRR23626001, SRR23626002, SRR23626003, SRR23626004, SRR23626005, SRR23626006, SRR23626007, SRR23626008, SRR23626009, SRR23626010, SRR23626011, SRR23626012, SRR23626013, SRR23626014, SRR23626015, SRR23626016, SRR23626017, SRR23626018, SRR23626019, SRR23626020, SRR23626021, SRR23626022, SRR23626023, SRR23626024, SRR23626025, SRR23626026, SRR23626027, SRR23626028, SRR23626029, SRR23626030, SRR23626031, SRR23626032, SRR23626033, SRR23626034, SRR23626035, SRR23626036, SRR23626037, SRR23626038, SRR23626039, SRR23626040, SRR23626041, SRR23626042, SRR23626043, SRR23626044, SRR23626045, SRR23626046, SRR23626047, SRR23626048, SRR23626049, SRR23626050, SRR23626051, SRR23626052, SRR23626053, SRR23626054, SRR23626055, SRR23626056, SRR23626057, SRR23626058, SRR23626059, SRR23626060, SRR23626061, SRR23626062, SRR23626063, SRR23626064, SRR23626065, SRR23626066, SRR23626067, SRR23626068, SRR23626069, SRR23626070, SRR23626071, SRR23626072, SRR23626073, SRR23626074, SRR23626075, SRR23626076, SRR23626077, SRR23626078, SRR23626079, SRR23626080, SRR23626081, SRR23626082, SRR23626083, SRR23626084, SRR23626085, SRR23626086, SRR23626087, SRR23626088, SRR23626089, SRR23626090, SRR23626091, SRR23626092, SRR23626093, SRR23626094, SRR23626095, SRR23626096, SRR23626097, SRR23626098, SRR23626099, SRR23626100, SRR23626101, SRR23626102, SRR23626103, SRR23626104, SRR23626105, SRR23626106, SRR23626107, SRR23626108, SRR23626109, SRR23626110, SRR23626111, SRR23626112, SRR23626113, SRR23626114, SRR23626115, SRR23626116, SRR23626117, SRR23626118, SRR23626119, SRR23626120, SRR23626121, SRR23626122, SRR23626123, SRR23626124, SRR23626125, SRR23626126, SRR23626127, SRR23626128, SRR23626129, SRR23626130, SRR23626131, SRR23626132, SRR23626133, SRR23626134, SRR23626135, SRR23626136, SRR23626137, SRR23626138, SRR23626139, SRR23626140, SRR23626141, SRR23626142, SRR23626143, SRR23626144, SRR23626145 (Zygnema and Physcomitrium).
Raw metabolite profiling data are available on Zenodo: 10.5281/zenodo.10805605
Details on WGCNA are available on Zenodo: 10.5281/zenodo.14234484
Data can be interactively explored at https://rshiny.gwdg.de/apps/streptotime/

Research involving human participants, their data, or biological material

Policy information about studies with human participants or human data. See also policy information about sex, gender (identity/presentation), and sexual orientation and race, ethnicity and racism.

Reporting on sex and gender	n/a
Reporting on race, ethnicity, or other socially relevant groupings	n/a
Population characteristics	n/a
Recruitment	n/a
Ethics oversight	n/a

Note that full information on the approval of the study protocol must also be provided in the manuscript.

Field-specific reporting

Please select the one below that is the best fit for your research. If you are not sure, read the appropriate sections before making your selection.

☒ Life sciences ☐ Behavioural & social sciences ☐ Ecological, evolutionary & environmental sciences

For a reference copy of the document with all sections, see nature.com/documents/nr-reporting-summary-flat.pdf

Life sciences study design

All studies must disclose on these points even when the disclosure is negative.

Sample size	Sample sizes (for RNA-Seq as well as the other experiments) were chosen based on the best practices of the field (at least 3 biological replicates per condition). Each analysis involved millions of pooled cells, all can be assumed to behave similarly (as they were vegetative cells from the same starting culture). For each species, at minimum 90 samples for RNAseq and physiological data points were analysed. Sequencing was then performed to a depth that was chosen based on approaching saturation level (based on obtaining differential expression patterns among the given number of genes). All data were used except for an additional set of samples that turned out to be irrelevant for the study (constant light, only acquired for Me). The experiments were not randomized. The Investigators were not blinded to allocation during experiments and outcome assessment.
Data exclusions	No data were excluded.
Replication	For the physiological, metabolite, and morphological analysis as well as RNAseq, at least three independent experiments were conducted (at different time points).<br>The stress experiments for all organisms were performed in independent biological triplicates. Following the start date of stress exposure is indicated.<br><br>Me: Cold-stress: 19/10/21; 21/10/21; 26/10/21<br>Heat-stress: 11/11/21; 16/11/21; 18/11/21<br>High light: 23/11/21; 25/11/21; 30/11/21<br>Addional Control-timeline: 9/11/21; 7/12/21; 9/12/21<br><br>Zc: Cold-stress: 27/04/22; 05/05/22; 30/05/22<br>Heat-stress: 27/04/22; 05/05/22; 30/05/22<br>High light: 04/04/22; 07/04/22; 11/04/22<br>Addional Control-timeline: 27/04/22; 05/05/22; 30/05/22<br><br>Pp: Cold-stress: 21/02/22; 24/02/22; 28/02/22<br>Heat-stress: 19/04/22; 20/04/22; 21/04/22<br>High light: 04/04/22; 07/04/22; 11/04/22<br>Addional Control-timeline: 21/02/22; 24/02/22; 28/02/22<br><br>All attempts were successful.
Randomization	All experiments are based on a random selection of millions of cells from a minimum of three pooled cultures on agar plate per replicate. The start culture was one homogenous culture that was equally distributed to inoculate the plates. A random selection of millions of cells thus ended up in one plate that was exposed to a certain condition. Prior to start, all plates were thus equal.
Blinding	Blinding was not relavant for this study. It is irrelevant for the bioinformatics because we worked with all versus all comparisons, unsupervised methods and all pipelines are fully transparent on GitHub. All cell-based evaluation is quantifyable and unambigous. Further, the information is fully provided and re-evaluatable.

Reporting for specific materials, systems and methods

We require information from authors about some types of materials, experimental systems and methods used in many studies. Here, indicate whether each material, system or method listed is relevant to your study. If you are not sure if a list item applies to your research, read the appropriate section before selecting a response.

Materials & experimental systems

n/a	Involved in the study	
☒	☐	Antibodies
☐	☒	Eukaryotic cell lines
☒	☐	Palaeontology and archaeology
☒	☐	Animals and other organisms
☒	☐	Clinical data
☒	☐	Dual use research of concern
☐	☒	Plants

Methods

n/a	Involved in the study	
☒	☐	ChIP-seq
☒	☐	Flow cytometry
☒	☐	MRI-based neuroimaging

Eukaryotic cell lines

Policy information about cell lines and Sex and Gender in Research

Cell line source(s)	Mesotaenium endlicherianum (Zygnematophyceae, Streptophyta): The alga has the collection number SAG 12.97. The biomaterial provider is the Experimental Phycology and Culture Collection of Algae in Göttingen, Germany. The alga was originally isolated in Portugal, Quiaios, Lagoa das Bracas, plankton, Lat./Long.(Precision): 40.243191 / -8.80488.<br>Zygnema circumcarinatum (Zygnematophyceae, Streptophyta): The alga has the collection number SAG 698-1b. The biomaterial provider is the Experimental Phycology and Culture Collection of Algae in Göttingen, Germany. The alga was originally isolated in Bohemia, from a ditch at meadow Poselteich (Poselský rybnik) near Hirschberg (Doksy)<br>Lat. / Long. (Precision): 50.552702 / 14.669362 (800m)
Authentication	Authentication was carried out directly by the biomaterial provider, the Experimental Phycology and Culture Collection of Algae in Göttingen, Germany, via microscopy and genetic markers.
Mycoplasma contamination	n/a
Commonly misidentified lines<br>(See ICLAC register)	n/a

Dual use research of concern

Policy information about dual use research of concern

Hazards

Could the accidental, deliberate or reckless misuse of agents or technologies generated in the work, or the application of information presented in the manuscript, pose a threat to:

No	Yes	
☒	☐	Public health
☒	☐	National security
☒	☐	Crops and/or livestock
☒	☐	Ecosystems
☒	☐	Any other significant area

Experiments of concern

Does the work involve any of these experiments of concern:

No	Yes	
☒	☐	Demonstrate how to render a vaccine ineffective
☒	☐	Confer resistance to therapeutically useful antibiotics or antiviral agents
☒	☐	Enhance the virulence of a pathogen or render a nonpathogen virulent
☒	☐	Increase transmissibility of a pathogen
☒	☐	Alter the host range of a pathogen
☒	☐	Enable evasion of diagnostic/detection modalities
☒	☐	Enable the weaponization of a biological agent or toxin
☒	☐	Any other potentially harmful combination of experiments and agents

Plants

Seed stocks	Physcomitrium patens Gransden 2004 strain 40001 protonema was used; the strain was obtained from the International Moss Stock Center (IMSC); the strain was orignally isolated in Gransden Wood, Cambridge (United Kingdom)
Novel plant genotypes	No novel genotypes were generated
Authentication	n/a
