# Supplementary material for: Time-resolved oxidative signal convergence across the algae–embryophyte divide
Source: Nat Commun. 2025 Feb 19;16:1780. doi: 10.1038/s41467-025-56939-y (PMC11840003; doi:10.1038/s41467-025-56939-y)
Supplement: Supplementary file 3 — Description of Additional Supplementary Files [file 41467_2025_56939_MOESM3_ESM.pdf]

<u>Description of Additional Supplementary Files</u>

Supplementary Data 1: Enriched GO terms among the significantly differentially expressed genes in 51 comparisons (stress versus control) represented in the three organisms.

Supplementary Data 2: Left: Modules (different color names) and numbers of genes therein recoverd by WGCNA. Right: clusters recovered by DPGP.

Supplementary Data 3: Presence / absence distribution of HOGs.
